# Enumeration and comprehensive in-silico modeling of three-helix bundle structures composed of typical αα-hairpins

**DOI:** 10.1186/s12859-021-04380-5

**Published:** 2021-09-27

**Authors:** Koya Sakuma, Shintaro Minami

**Affiliations:** 1 SOKENDAI, The Graduate University for Advanced Studies, 38 Nishigonaka, Myodaiji, Okazaki, 444-8585 Japan; 2 Institute for Molecular Science, 38 Nishigonaka, Myodaiji, Okazaki, 444-8585 Japan; 3 Independent Researcher, Seattle, WA USA

**Keywords:** α-Helical protein, Helical bundle, Protein design, Fragment assembly, αα-Hairpins

## Abstract

**Background:**

The design of protein structures from scratch requires special attention to the combination of the types and lengths of the secondary structures and the loops required to build highly designable backbone structure models. However, it is difficult to predict the combinations that result in globular and protein-like conformations without simulations. In this study, we used single-chain three-helix bundles as simple models of protein tertiary structures and sought to thoroughly investigate the conditions required to construct them, starting from the identification of the typical αα-hairpin motifs.

**Results:**

First, by statistical analysis of naturally occurring protein structures, we identified three αα-hairpins motifs that were specifically related to the left- and right-handedness of helix-helix packing. Second, specifying these αα-hairpins motifs as junctions, we performed sequence-independent backbone-building simulations to comparatively build single-chain three-helix bundle structures and identified the promising combinations of the length of the α-helix and αα-hairpins types that results in tight packing between the first and third α-helices. Third, using those single-chain three-helix bundle backbone structures as template structures, we designed amino acid sequences that were predicted to fold into the target topologies, which supports that the compact single-chain three-helix bundles structures that we sampled show sufficient quality to allow amino-acid sequence design.

**Conclusion:**

The enumeration of the dominant subsets of possible backbone structures for small single-chain three-helical bundle topologies revealed that the compact foldable structures are discontinuously and sparsely distributed in the conformational space. Additionally, although the designs have not been experimentally validated in the present research, the comprehensive set of computational structural models generated also offers protein designers the opportunity to skip building similar structures by themselves and enables them to quickly focus on building specialized designs using the prebuilt structure models. The backbone and best design models in this study are publicly accessible from the following URL: https://doi.org/10.5281/zenodo.4321632.

**Supplementary Information:**

The online version contains supplementary material available at 10.1186/s12859-021-04380-5.

## Background

Designing protein structures from scratch requires the careful selection of the length and types of the secondary structures and the loop types; further, the global structure and the local structural motifs need to be consistent. However, it is difficult to predict the combinations of building blocks that result in globular and protein-like conformations without simulations. Therefore, it would be beneficial for protein designers to limit the building blocks to the typical ones and enumerate the dominant subsets of their possible combinations in order to find promising combinations that result in highly designable backbone structures. In addition, once such conformational enumeration is performed, their results can be shared with other designers, and would enhance further design studies by allowing them to skip resampling the similar structures.

The αα-hairpin is a well-known structural motif, which consists of two adjacent α-helices and a loop region in between [1]. The loop region allows two flanking α-helices to pack into antiparallel arrangements, and the steep turn leads to tight non-local contacts between the two adjacent α-helices. Although the loop regions in general show non-repetitive structures and their conformations are more complicated than secondary structures, a few of them show clear patterns and can be classified into several subtypes, and are thus regarded as local motifs [2, 3]. Several pioneering studies have identified certain typical conformations of loops that are specifically related to αα-hairpins [1, 4, 5] and utilized them for design [6].

In this study, we considered single-chain three-helix bundles as the simplest tertiary structures and investigated the conditions required to consistently construct them. The single-chain three-helix bundle is composed of three α-helices and two connecting loop regions that fold into hairpin conformations, causing neighboring α-helices to pack tightly into a compact antiparallel bundle configuration. Consequently, the third α-helix can be packed parallel to the first helix. The single-chain three-helix bundle structures are frequently observed in naturally occurring proteins and also have been designed artificially as well [7, 8]. Of note, the design of the single-chain three-helix bundle was one of the earliest efforts in the de novo protein design [8]. The design of helical bundles or mutiple-chaine coiled-coils is nowadays one of the largest fields in the protein design study, allowing diverse α-helix arrangements [9]. It is now clear what residue-residue non-local interactions can cause tight packing between α-helices [10] and result in various helix-bundle arrangements [11], which originates from analysis and design of coiled-coil structures [12–14]. Such knowledge for helical bundle designs have recently led to design of antibody-like and interleukin-mimicking artificial proteins [15–17] and programmable heterodimers [18]. However, many of previous works focus on the interface design between α-helices and do not pay much attention to the detailed conformations of loops that optimally connect individual α-helices. Therefore, when compared to coiled-coils and peptide assemblies that are composed of several independent chains, it still remains unclear and undocumented which combinations of the α-helix lengths and loop types result in compact single-chain helical bundle structures. Understanding the dominant subsets of possible conformational spaces allowed for single-chain helical bundles will be fundamentally important and even informative to efficiently design pharmacologically valuable artificial proteins. To this end, we aimed to understand which combinations of αα-hairpins and α-helix lengths can result in compact single-chain three-helical bundle structures, considering the αα-hairpins as the fundamental building blocks.

## Results and discussion

### Specific αα-hairpin loops determine the handedness of helix-helix packing

To identify typical hairpin motifs, we performed a statistical analysis of helix-loop-helix fragments and found that shorter loops are present in greater frequency (Additional file 1: Figure S1). To focus on hairpins rather than general helix-loop-helix fragments, we defined helix-orientation vectors [19] and calculated their crossing angles (Additional file 1: Figure S1). On applying the condition that the helix-helix crossing angles θ_HH_ are less than 60° in the fragment dataset, we found a decrease in the population of single-residue loops, as the short helix-loop-helix prefers corners or kinks rather than hairpins. We focused on the more frequent short hairpin fragments and extracted 2, 3, and 4 residue length loops for subsequent analysis.

**Fig. 1:**
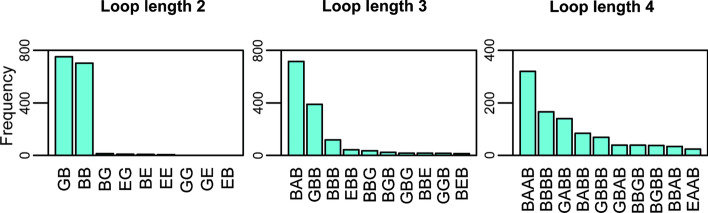
Identification of typical αα-hairpin motifs by the ABEGO representation for the loop conformations. The distributions of the top-10 typical hairpin conformations for two, three, and four-residue length loops that were identified by ABEGO. We can see several specific series of backbone torsion angles are strongly preferred in the hairpin loop region

To investigate the preferable loop conformations related to the specific handedness of helix-helix packing in naturally occurring protein structures, we assigned a 5-state coarse-grained representation for the backbone torsion angle, i.e., ABEGO representations (Additional file 1: Figure S2) for each fragment and evaluated their statistical information [2]. ABEGO is a five-state coarse-grained representation of polypeptide backbone dihedral angles; Ramachandran map is divided into four sections and labelled by single letters A, B, E and G, to enable the representation of dihedral angle series by character strings. The A region roughly corresponds to the conformation of α-helix, and the B region corresponds roughly to the β-strand conformation. The G region corresponds to left-handed α-helix, and the E region represents the rest of the Ramachandran map. The O state corresponds to the cis-conformation of peptide bond, which are almost negligible in this paper. Then we sorted the backbone torsion types specified by the ABEGO representations by their population and found that the hairpins showed limited conformations (Fig. 1). For example, the GB and BB loops occupied more than 90% of the top five frequent populations among two-residue loops.

**Fig. 2:**
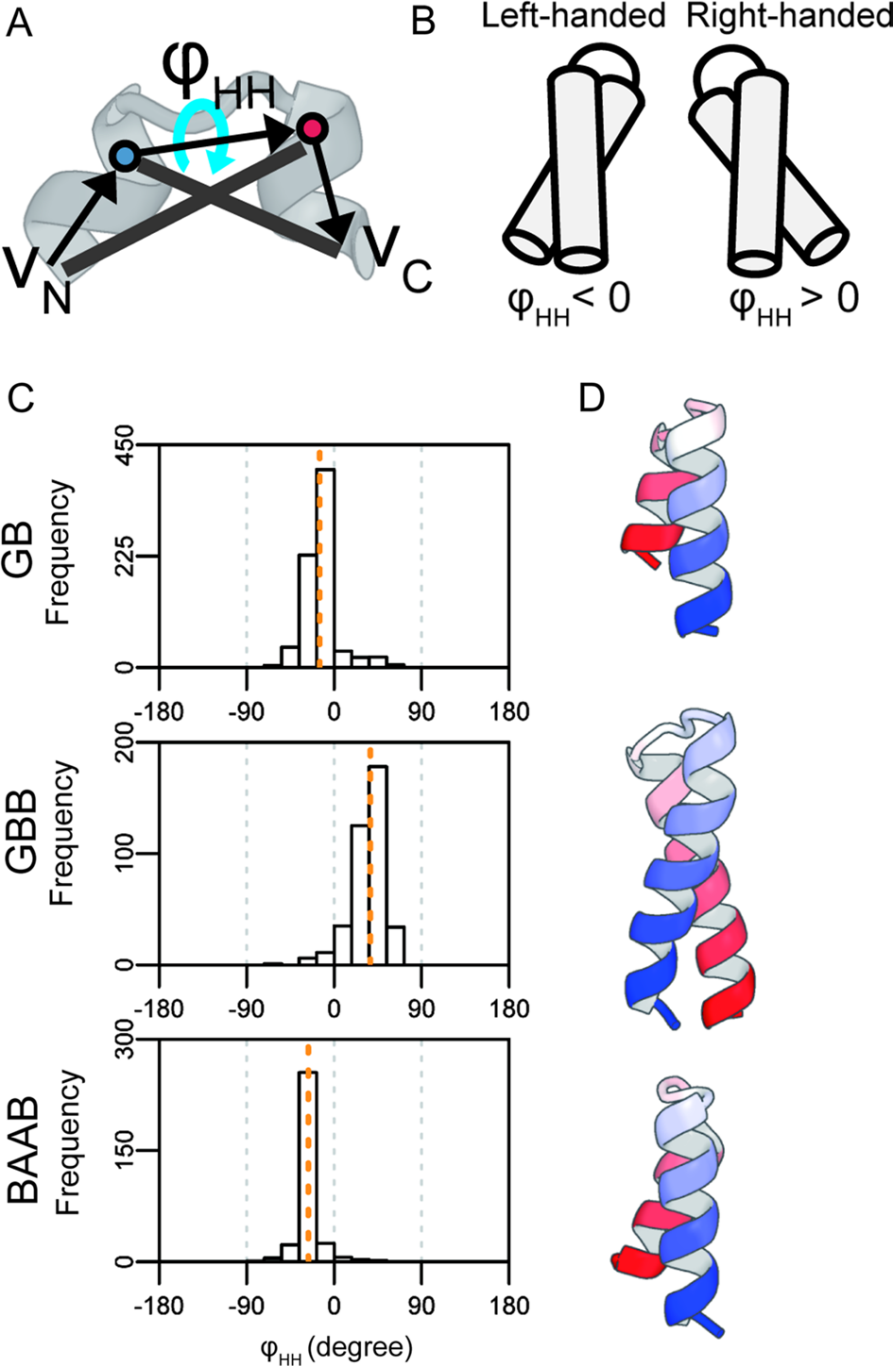
The handedness of helix-helix packing forced by typical αα-hairpin motifs. **A** Definition of φ_HH_ **B** Definition of packing handedness **C** The distribution of φ_HH_ for GB, GBB, and BAAB hairpins. The horizontal axis represents the values of the helix-helix dihedral angle φ_HH_, and the vertical axis corresponds to the number of loop fragments in the dihedral bins. The barplots are binned every 18°, and the orange dotted line indicates the median value of the distribution. **D** Representative structures for the GB, GBB, and BAAB hairpins. The structures show the GB and BAAB hairpins are related to left-handed packing, and GBB is related to right-handed packing. The α-helices are shown as cartoons and are colored in the blue-white-red gradient from the N to the C-terminus

To identify hairpins that show specific handedness in helix-helix packing, we defined helix-helix dihedral angles φ_HH_ and calculated the ratio of left- (L-) and right- (R-) types among the helix-helix dihedral angle distribution (Fig. 2). We selected the most populated loop types that showed R/L or L/R ratios higher than 5.0 in each class of loop lengths as representative hairpin species. This resulted in the selection of the GB, GBB, and BAAB loops; we did not select BAB loop because their inter-helix dihedral angles were broadly distributed, resulting in both left- and right-handed helix-helix packing (Additional file 1: Figure S3).

We extracted the structures whose φ_HH_ was nearest to the median of the angle distribution as the class representatives. The representative structure and the distribution of φ_HH_ clarified that GB loops are closely related to the L-type handedness of helix-helix packing motifs (Fig. 2). Similarly, the GBB loop was related to the R-type and the BAAB loop to L-type packing. The handedness of helix packing for the GB, GBB, and BAAB loops was broadly consistent with a previous report [20] and the overall tendency did not change when we performed the same analysis for different dataset (Additional file 1: Figure S4). We also checked the clustering quality by sequence alignments for each hairpin structure, and observed typical periodic patterns of hydrophobic residues in the flanking helix regions (Additional file 1: Figure S5). We concluded that classification using the ABEGO patterns worked well to extract hairpin motifs related to the specific handedness of helix-helix packing.

Previous studies on αα-hairpins reported both L and R types of helix-helix packing can result from GB or GBB hairpins [1, 4]. However, we observed that these loops indeed strongly bias the handedness of helix-helix packing. This does not imply that a single ABEGO-level representation can always specify the single handedness of helix-helix packing; for example, BAABB loop can result in both the L and R type packing (Additional file 1: Figure S3). However, certain hairpin conformations such as GB, GBB, and BAAB can strongly determine the handedness of the packing of two flanking α-helices, and are an example of a pair of local and nonlocal structural motifs that are consistently incorporated into a single tertiary structure. As the spatial arrangements and orientation of two α-helices connected by a loop region are stereochemically determined by the backbone dihedral angles in the loop region, preference to specific handedness of helix-helix packing can be attributed to the rigid conformation of loop region. Hydrogen bond analysis using DSSP [21] revealed that intra-loop backbone-backbone hydrogen-bond network energetically stabilizes such typical loop conformations, making the loop conformation rigid enough to relate the local conformation of loops to the specific geometry helix-loop-helix fragments (Additional file 1: Figure S6–S8).

### Sequence-independent backbone-building simulations clarify the condition for building compact single-chain three-helix bundles

The length of the second α-helix is expected to play a crucial role in the construction of compactly packed single-chain three-helical bundle structures since extension of α-helix leads to large repositioning of the following segments. As one turn of the α-helix requires 3.6 residues, compact bundle structures may appear for every increase of 3 or 4 residues. However, it remains unclear which exact combination of loops and helix-length results in a compact single-chain three-helix bundle structure. Therefore, we performed comparative sequence-independent fragment-assembly simulations to identify which combination of loops and helix lengths result in tight packing between the first and third α-helices.

The set of backbone dihedral angles of the fragments are roughly specified in the ABEGO representation (referred to as “blueprint” [22]) and are used in fragment-picking before the fragment-assembly simulations. Hereafter, we refer to this type of fragment assembly simulation guided by the blueprints as backbone-building simulations. Using the GB, GBB, and BAAB loops identified in the previous section, we constructed blueprint files for various types of single-chain three-helix structures and systematically scanned the length of the second α-helix. Next, 2500 trajectories of backbone-building simulations were performed for each of these blueprints [23, 24]. We prepared ideal single-chain three-helical bundle decoys using CC-builder for the reference structures [25], and calculated the template-match score (TM-score) of the final structure from each trajectory that was referenced by the decoys to quantify the success ratio of the backbone-building simulations [26]. Importantly, we had two reference decoys for each blueprint-based folding simulation because single-chain three-helical bundles can take two types of helix configurations; i.e., a clockwise (CW) or counter-clockwise (CCW) arrangement of three α-helices (Additional file 1: Figure S9).

**Fig. 3:**
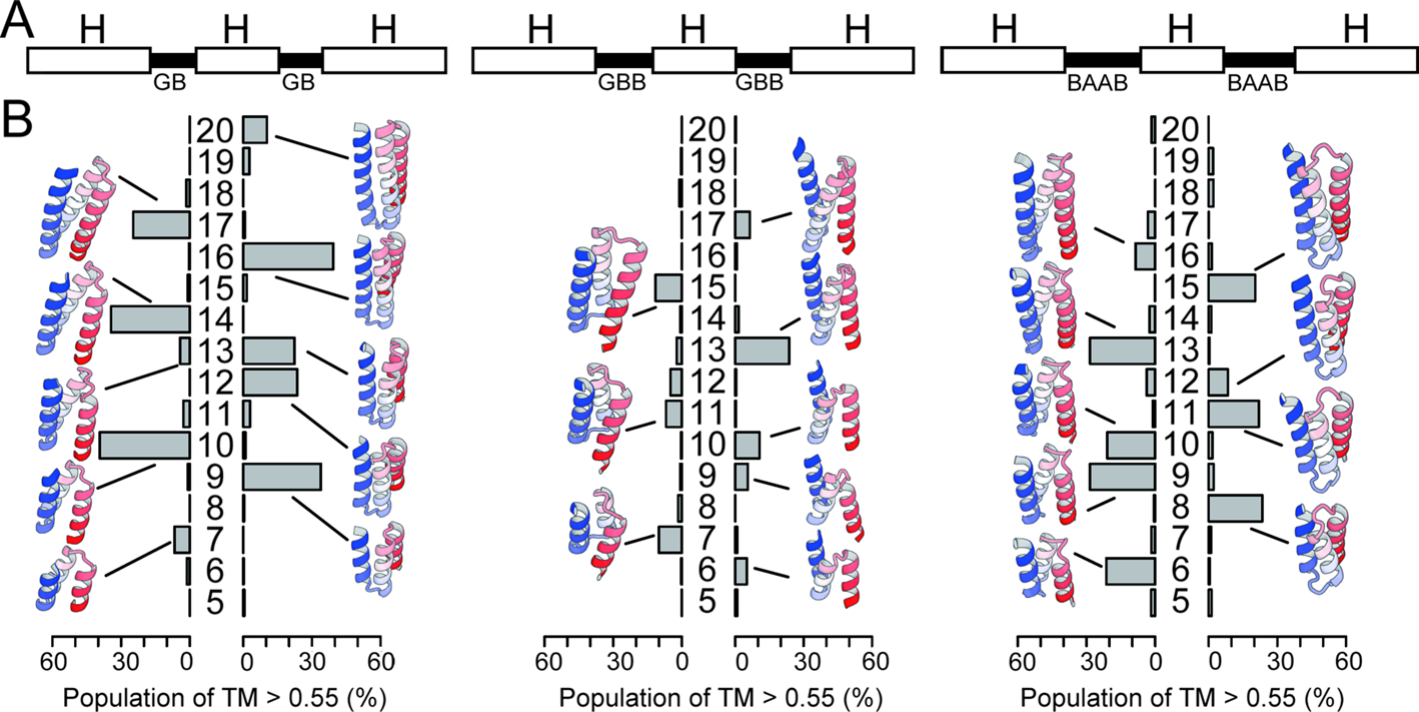
The length of the second helix is highly responsible for the compaction of single-chain three-helix bundles. (Top) The blueprints of single-chain three-helix bundles. The white bars indicate the α-helices, and the black bars indicate the loop regions, where integer H denotes the variable length of the second α-helix. The alphabets beneath the loop represent the ABEGO of the loop region specified in the blueprint. (Bottom) Bar graphs to summarize the foldability of each blueprint with the variable length of the second α-helix, H, scanned from 5 to 20 residues.The bars represent the population of folded structures that showed a TM-score higher than 0.55 as referenced by ideal CW (left) or CCW (right) three-helical topologies. The vertical axis represents the length of the second α-helix H. The structures shown beside the bars are the representative snapshots from the backbone-building simulations with highest TM-scores

The results of the backbone-building simulations are summarized in Fig. 3. The simulations showed three important features that are summarized here by taking the results for the helix-GB-helix-GB-helix simulations as examples. First, the length of the second alpha-helix plays a crucial role in the construction of compactly packed single-chain three-helical bundle structures; the success ratio of the backbone-building simulations was obviously related to the periodicity of the α-helix structure. For example, CW bundles can be efficiently generated with the second α-helix with lengths of 10, 14, and 17 residues for helix-GB-helix-GB-helix blueprints. Similarly, the second α-helix with lengths of 9, 12, 16 residues resulted in CCW bundles. Here, the peaks were separated in every three or four residues, which was consistent with the canonical α-helix structure that requires 3.6 residues per turn. Second, certain combinations of loops and helix-lengths do not yield well-packed helix bundles. For example, the blueprint with a 15 residue helix in the middle cannot fold into a compact helical bundle. This is because the number of turns in the second α-helix is unable to pack the first and third helices closely, causing them to be apart from each other (Additional file 1: Figure S10). Such a blueprint has a local conformation that is inconsistent with the global structure of compact single-chain three-helical bundles. Third, the position of the peaks oscillates between CW and CCW bundles as the length of the second helix increases. The switch between a CW bundle to the neighboring CCW bundle is very sharp and sometimes requires an increase/decrease of a single residue. For example, the blueprint with a 16-residue helix in the middle preferentially results in the CCW bundle structure, and an increase of one residue results in a preference for the CW bundle. Overall, the results of the backbone-building simulations agree with the qualitative expectations that were guided by the periodicity of the α-helix structure, and provide further detailed information on the exact combination of loop types and helix-lengths that result in compact bundle conformations. These results were not affected when different threshold and reference structures were used for analysis (Additional file 1: Figures S11 and S12).

Taken together, these results indicate that the appropriate combination of local loop motifs and the length of the secondary structures are relatively rare among the possible combinations, especially under approximation that the loops and α-helices are semi-rigid under ABEGO constraints on backbone dihedral angles. In our simple simulations for single-chain three-helix bundle structures, approximately half of the blueprints were able to generate compact bundle conformations. As the foldable combinations of building blocks are rare and sparsely distributed even for simple single-chain three-helical bundles, valid combinations for more complicated topologies are expected to become rarer and more difficult to find. We expect that the possibility of obtaining foldable combinations will decrease exponentially as the number of secondary structures increases, and it will be difficult to hypothesize as to which combinations may result in a compact, globular, and protein-like structure without exhaustive sampling in the conformational space.

Other types of blueprints, such as for helix-GBB-helix-GBB-helix, and helix-BAAB-helix-BAAB-helix showed results similar to the GB-blueprint. Interestingly, the “phase” of the peak oscillation was inverted between the GBB and BAAB-blueprints, whereas the positions of the peaks were similar to each other, reflecting the local handedness of the hairpin structures. The former results in a CCW bundle when the second helix has 13 residues, and the latter yields a CW bundle in the same conditions. These observations that the local handedness of hairpins can control the global chirality of the topology may be informative for efficiently diversifying the shapes of design proteins. Additionally, the blueprints showing a mixture of hairpins with different handedness failed to pack the first and third α-helices because their crossing angles do not cancel out (Additional file 1: Figure S10 and S13–17).

### Amino acid sequence design suggests that the enumerated globular single-chain three-helix bundle structures may be designable

**Fig. 4:**
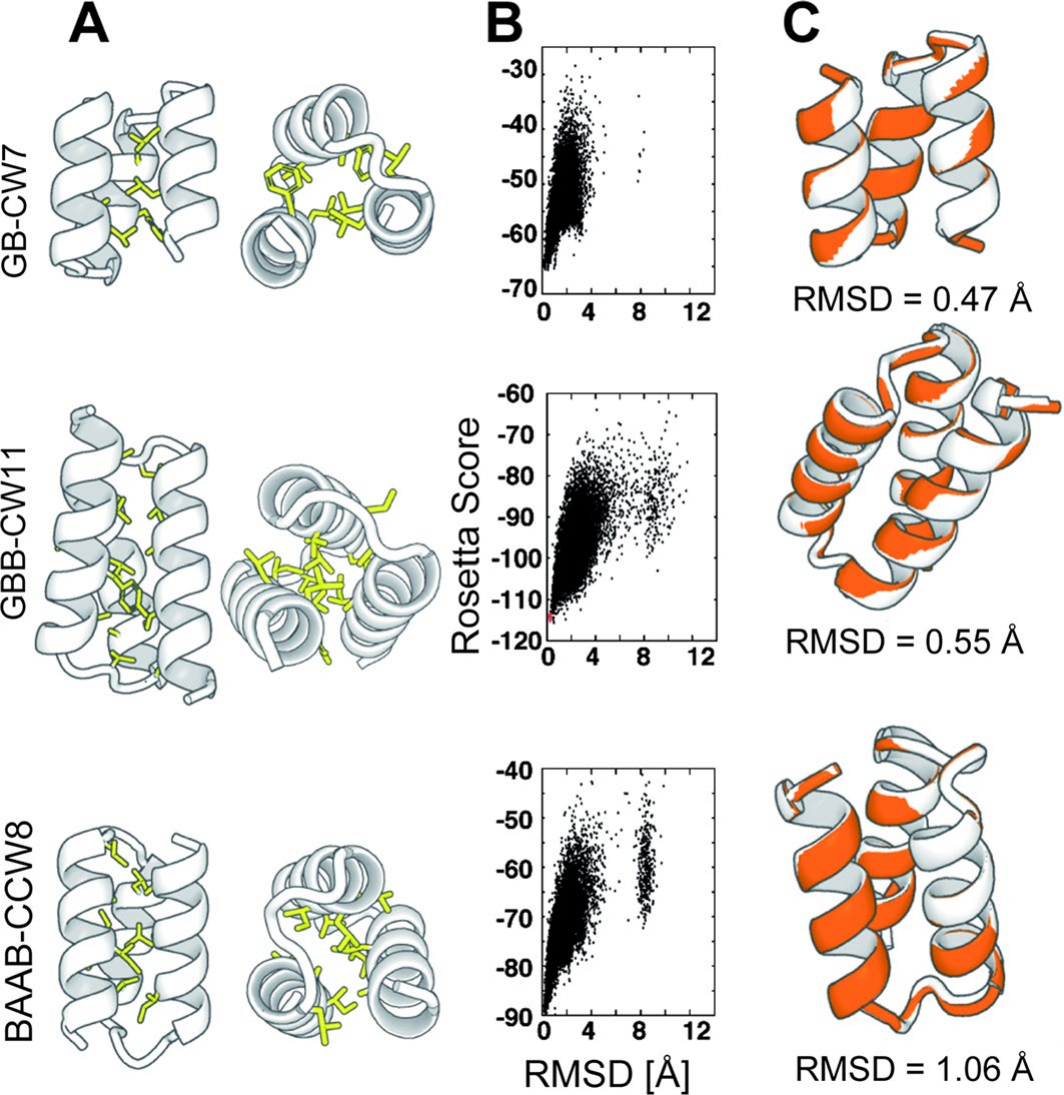
The representative structures and the results of sequence-dependent folding simulations of the designed single-chain three-helix bundles: GB-CCW9, GBB-CW11, and BAAB-CCW8. **A** The side-view and top-view of the designed structures with the α-helix shown as a cartoon and the hydrophobic side-chains represented as sticks. **B** The results of the folding and relax simulations. The vertical axis represents the Rosetta score, and the horizontal axis represents the root-mean-square deviation from the target structures. The black dots correspond to the final snapshots of the fragment-assembly folding simulations starting from extended conformations, and the red dots correspond to the final snapshots of the relax simulations starting from the native conformations. These designs are predicted to fold into the target topologies because the trajectories of the folding simulations can reach the near-native ensembles. **C** The lowest score models in folding simulations (orange) superimposed onto the design models (white). The predicted models and design models agrees well, which suggests the designed amino acid sequences fold well into the target conformations

As the backbone model generated in the previous section lacked any information on amino acid sequences, we performed sequence designs using Rosetta [24] to check if the compact single-chain three-helix bundle structures are designable as concrete amino acid sequences. We selected 27 backbone structures that are listed in Fig. 3 and performed amino acid sequence designs for these backbone structures. We designed ~ 7000–9000 sequences for each backbone structure and observed that the interfaces between the first and third α-helices recovered the sequence motifs for helix-helix packing (Additional file 1: Figure S18). The results show that the relative arrangements of the first and third α-helices sampled in the sequence-independent backbone-building simulations are realistic enough to mold the typical amino acid sequences observed in helix-helix packing motifs. The optimal combinations of local properties such as the hairpin types and α-helix lengths lead to the successful recovery of non-local features. From these ensembles of design models, we selected the most foldable sequences for each topology using sequence-dependent fragment assembly simulations [27] (Fig. 4 and Additional file 1: Figure S19–S21). In most of the simulation settings, the lowest-score models agreed well with the design models and recovered local hairpin structures well (Additional file 1: Figure S22–S27 and Table S1). We also performed negative-control designs in which the loop regions of the up-down helix bundles have atypical conformations, such as EE, BEB, and BEEE. The best-effort designs for these backbone models were indistinguishable in terms of per-residue Rosetta scores from the designs with typical hairpin motifs (Additional file 1: Table S2). However, they were not able to efficiently fold into the target topology in the sequence dependent fragment-assembly simulations (Additional file 1: Figure S28). This result suggests that the compact up-down bundle structures with typical hairpins have higher designability than the ones composed of atypical hairpins. For these best design models, we performed blast search using blastp against a non-redundant sequence database [28, 29] and confirmed that three were no similar sequences found in the database.

To confirm the stability of designed proteins independently from the Rosetta score function and fragment assembly simulations, we utilized molecular dynamics simulations of designed proteins models and assessed their quality [30, 31]. We performed molecular dynamics simulations for the 27 best design models using GPU-accelerated GROMACS 2020.6 [32, 33] alongside Amber 15FB force field [34]. For each design, we performed 10 trajectories of 100 ns molecular dynamics simulations with explicit TIP3P water models. After energy minimization and equilibration, we performed 100 ns of the production run under the pressure of 1 bar and temperature of 300 K. The simulation showed the most of the design models can stay within 5 Å in the root-mean-square deviations (RMSD) of Cα coordinate from the designed structures for 100 ns (Additional file 1: Figure S29–S34). The only exceptions were 7th trajectory of GB-CW7 and 3rd and 8th trajectories of GBB-CCW6 (Additional file 1: Figure S29 and S31), which resulted in partial unfolding of the structures. These structures may be unstable probably because they have too small hydrophobic cores to maintain the designed topologies. Overall, the molecular dynamics simulations showed that the designed proteins were stable enough to keep the native conformation in the solution state. These results provided independent validation for the designability of the backbone structures we sampled by fragment assembly with the Rosetta score function.

As we designed up-down types of helical bundles, whose substructures can be regarded as antiparallel and parallel coiled coils, we confirmed whether the best-designed sequences can be recognized as coiled-coils by DeepCoil [35–37]. Interestingly, the predicted probability to observe coiled-coil arrangements within the designed structure increased as the length of the design proteins increased (Additional file 1: Figure S35–S40). Approximately, when the length of the second α-helix is longer than 15 amino-acid residues, the probability to recognize the sequence as coiled-coil became higher than the significance threshold. This suggests that these up-down helical bundles can be regarded as coiled coils when the chain lengths are large enough. Therefore, although we designed amino-acid sequences without considering the structures are related to coiled coils, sequence design techniques for coiled coils can be repurposed to the sequence design of large helical bundles and may result in more optimized helix-helix packing, which may lower the computational cost of design and improve the yield of successful design sequences. On the other hand, smaller helical bundles failed to be predicted as coiled coils. This does not immediately imply that such small helical bundles are not designable; such small helical bundles were designed in a previous study [38]. Therefore, the sequence design of such small helical bundles should be performed without considering the structure as coiled-coils. This analysis suggested that we may be able to select optimal design methods depending on the size of target helical bundles. It is also interesting whether parametrically designed multiple-chain coiled coils can be redesigned into single-chain helical bundles by designing the loops connecting the α-helices; the question is whether the designer can find appropriate loop conformations to connect the α-helices [39] without frustrations between local and nonlocal interactions [40, 41].

Finally, to detect knobs-into-holes structure in our designs, we performed structure analysis using SOCKET [42]. According to SOCKET, knob-into-holes structures were observed roughly in two-third of our designs (Additional file 1: Figure S41–S44). In addition, SOCKET detected coiled-coil structure in 4 of our designs, although we did not intend to design coiled-coil-like substructures in our design scheme. This also suggests that the design of helical bundles shares many similar aspects with the design of coiled coils, and the rich and matured protocols for coiled-coil design can be imported into the design of single-chain helical bundles.

## Conclusion

In this study, we used single-chain three-helix bundles as simple models of protein tertiary structures and investigated the conditions required to construct them, and aimed to understand the mechanisms by which these local and nonlocal motifs are consistently incorporated into a single three-dimensional structure. First, we showed that the GB- and BAAB-hairpins are related to left-handed helix-helix packing, whereas the GBB-hairpins are related to right-handed packing. Second, by enumerating the combinations of the hairpin types and the helix length, we identified the combinations of helix-length and loop types that resulted in successful compaction of single-chain three-helix bundle structures. As we have enumerated most of the backbone structures that are potentially obtainable for these simple topologies under the condition that the hairpins are limited to GB, GBB, or BAAB, and the lengths of the second α-helix are less than 20 residues, no other single-chain up-down three-helical bundle structures are plausible in this subspace of the structural space. Combined with the observation that the populations of loops are strongly biased towards a limited number of typical conformations, such enumeration can cover most of the possible conformational space.

We also showed that the backbone structures composed of such short hairpin motifs may be highly designable by amino acid sequence design and sequence-dependent folding simulations, although experimental validation for these designed proteins should be done elsewhere. In addition, Molecular dynamics simulations supported that the designed proteins are stable in solution, which suggests that designed proteins do not have internal frustrations between local and nonlocal interactions. Using programs to detect coiled-coil sequences and structures, we also found that the designed sequences and structures can be recognized as coiled-coil when the sequences are long enough. This implies sequence design methods based on sequence periodicity of coiled coils, which is usually utilized in design of multi-chained coiled coils or peptide assemblies, can be repurposed for the design of single-chain up-down helical bundles to realize optimized helix-helix packing.

Though our analyses are limited to the simplest class of tertiary structure, single-chain up-down three-helical bundles, we have shown that the enumerative exploration into the conformational space can clarify the appropriate combinations of building blocks. We also showed such exploration can yield transferable structural resources for protein design that can be shared with other protein designers. As such enumeration does not need to be done twice, data sharing among designers would promote advances in the protein design fields. To this end, the 27 types of backbone structure that we enumerated and the best sequences that we designed are now publicly available at https://doi.org/10.5281/zenodo.4321632.

## Method

### Initial dataset preparation

We composed a subset of the ECOD database (version 238) whose sequence redundancy was reduced by 40% sequence identity [43]. Next, secondary structures were assigned using DSSP [21], and a total of 39,938 helix-loop-helix substructures were extracted having loop lengths that were less than or equal to 10. We discarded the structures whose α-helices have less than or equal to 9 residues.

We prepared another dataset of PDB structures whose sequence redundancy was reduced by 25% sequence identity with resolution lower than 3.0 A using Pisces server [44], and obtained 29,149 helix-loop-helix structures. These structures were used to check the effect of resolution cut-off for the geometric analysis of helix-loop-helix fragments (Additional file 1: Figure S4).

### ABEGO-level dataset preparation

The backbone dihedral angles were translated into 5 state coarse-grained ABEGO representations (Additional file 1: Figure S2). ABEGO is a coarse-grained representation of polypeptide backbone dihedral angles, where the Ramachandran map is divided into four sections and labeled by single letters A, B, E and G. The O state corresponds to the cis-conformation of the peptide bond, which is almost negligible in this paper. The A region roughly corresponds to the conformation of α-helix, and the B region corresponds roughly to the β-strand conformation. The G region corresponds to left-handed α-helix, and the E region represents the rest of the Ramachandran map.

As it is ambiguous whether the dihedral angle of A in ABEGO representation is a loop region or α-helix termini, we removed the loop fragments that start/end with A of ABEGO. We only included fragments that started/ended with B, E, and G. After this data pruning, we obtained 19,844 helix-loop-helix fragments. We checked that the removal of fragments that started/ended with A did not largely change the distribution of the frequent loop types (Additional file 1: Figure S45). All of the date processing was performed with in-house R and python programs.

### Definition of the geometrical features of helix-loop-helix fragments

For the final and first single turn on the N/C-terminal α-helices of the helix-loop-helix fragments, the vectors ***v***_N_ and ***v***_C_ representing the orientation of these α-helices were defined as per Krissinel and Henrick [19]. Additionally, we defined the loop orientation vector ***v***_L_ as starting from the final/first Cα coordinates of the N-/C-terminal α-helices. Next, we defined 2-geometric features using ***v***_N,_***v***_C_, and ***v***_L_: (1) the helix-helix crossing angle θ_HH_, (2) the helix-helix dihedral angle φ_HH_. θ_HH_ is the crossing angle between two N/C-terminal α-helices (Additional file 1: Figure S1) defined by the arc-cosine of the inner-product of ***v***_N,_ and ***v***_C_. φ_HH_ is the inter-helix dihedral angle between two α-helices defined by ***v***_N_, ***v***_L_, and ***v***_C_ (Fig. 2). As we focused on αα-hairpins, we only collected fragments that satisfied the condition that θ_HH_ was less than 60° before performing the rest of the analysis.

### Sequence-independent fragment-assembly simulations: Backbone-building simulations

Sequence-independent fragment assembly simulations, which we referred to as backbone-building simulations, were performed using Rosetta BluePrintBDR [24] similarly as in Lin et al. [23]. The blueprint files were generated manually and were used in fragment picking to specify the backbone torsion in the ABEGO representation. For each site of proteins, 200 fragments were picked from the default structure library. For each blueprint, simulations were repeated for 2500 trajectories, and the final snapshots from the trajectories were used for structural analysis. A parameter set, fldcen.wts, was used as weight parameters for BluePrintBDR simulations.

In the analysis, two ideal decoy structures of single-chain three-helical bundles were used as references to calculate the TM-scores using TM-align [26]. We prepared two types of reference decoys, i.e., clockwise (CW) and counter-clockwise (CCW) bundles originating from the ideal decoy structures that were generated by CC-builder [25]. The parameters for CC-builder were as follows; oligomeric state 3; radius 6.75, 8.1, 9.0, 9.9, and 11.25 for × 0.75, × 0.90, × 1.00, × 1.10, and × 1.25 radius variant of helix bundles; pitch 300; interface Angle 20. Based on these decoys built by CC-builder, we manually modified their helix-orientation to up-down-up and packing chirality by re-sorting and mirroring the Cα coordinate and superimposing ideal α-helices onto the mirrored helix arrangements (Additional file 1: Figure S9). In the data analysis, the snapshots showing TM-scores higher than 0.55 were counted as folded into three-helical bundle structures [45]. To check the robustness against the change in reference structures, we systematically modified the diameter of reference helix-bundles by the magnitude of 0.75, 0.9, 1.10, and 1.25. We also changed the threshold of TM-score (0.50, 0.55, and 0.60) to check the robustness of the results. These parameters were found not to largely change the results (Additional file 1: Figure S11 and S12).

### Construction of negative-control helix-bundle structures

Based on the anti-parallel part of decoy structures described above, the loop region were modeled using Modeller [46] and six types of helix-helix hairpins that have atypical EE, BEB, and BEEE conformations were selected. Then the respective hairpin structures were repeated to form three-helix bundles composed of atypical hairpins. The severe steric clashes between alpha-helices were removed using Foldit-standalone [47].

### Amino acid sequence design and sequence-dependent folding simulations

Amino acid sequence design was performed using the Rosetta flxbb protocol [24] starting from the backbone structure that showed the best TM-score in the previous sequence-independent folding simulations. Score Talaris2014 was used in all designs and folding simulations including negative-control designs. In the loop region, amino acid profiles were constructed using similar loop structure fragments (RMSD < 2 Å) and used as constraints for residue types, similarly to Marcos et al. [48]. In addition, the specification on the residue types was refined based on the buriedness of the backbone atoms using in-house programs. The text files, i.e. the so-called “resfiles” specifying the final residues set were attached as supplementary files. We performed 10,000 design trials for each backbone model and obtained ~ 7000 to 9000 design sequences that passed the secondary structure filter. We selected the best 5–10 sequences using the fragment-quality score. We defined the fragment quality score as the average of the logarithm of the number of fragments with RMSD lower than 1.5 Å from the design model, similarly to Marcos et al. [48].

We performed sequence-dependent fragment-assembly folding simulations [27] to identify the best design sequences. Sequence dependent fragment assembly simulations, which we denoted “folding” simulations, were performed using AbinitoRelax binary in Rosetta suite with 200 3-mer and 9-mer fragments collected by psi-blast search in the default structure library. Near-native sampling simulations, which we denoted “relax” simulations, were performed by relax binary in Rosetta suite to sample near-native conformation starting from the designed structure models. 20,000 trajectories of fragment-assembly folding simulations were performed for each design protein, and their ability to fold into the target structures was evaluated by the shapes of the energy landscapes.

### MD simulations

All of the simulations were performed using GROMACS 2020.6 [32, 33] with Amber force field ff15FB [34]. First, we performed the in-vacuo energy minimization by the steepest descent for 500,000 steps, and the energy-minimized protein structures were solvated by TIP3P water models. The initial box size was set to 6 nm × 6 nm × 6 nm, which was large enough for all types of designs. The Na^+^ and Cl^−^ ions were introduced to the system at the concentration of 0.1 mol/L. Depending on the total charge of the designed proteins, additional Na^+^ or Cl^−^ ions were added to the system so that the system has zero net charges. The whole system was energy-minimized again for 500,000 steps.

With the step size of 2.0 fs, the whole system was equilibrated by 100 ps of NVT and NPT simulations under harmonic constraint for heavy atoms. Then 100 ns of production runs were performed without any external constraints to the system under 1 bar of pressure and 300 K of temperature. The production runs of the MD simulations were performed with LINCS constraint algorithm for the bonds between hydrogen atoms and heavy atoms. The temperature of the system was controlled to 300 K by the V-rescale algorithm (modified Berendsen thermostat) with the time constant of 0.1 ps. The pressure was controlled to 1.0 bar by Parrinello-Rahman algorithm with the time constant of 2 ps. The electrostatic part of the force field was calculated using the particle mesh Ewald scheme with the order of 4.

## Supplementary Information

**Additional file 1**. Supplementary Figures S1–S45, Supplementary Tables S1 and S2.

## Data Availability

The backbone models and best design models in this study are publicly accessible from the following URL: https://doi.org/10.5281/zenodo.4321632. The in-house python and R script for ABEGO analysis is disclosed in Github: For ABEGO-based analysis: https://github.com/yakomaxa/ssdoublet. Other scripts to reproduce the research are also disclosed in Github: For backbone building: https://github.com/yakomaxa/bbdesign_template. For MD simulations: https://github.com/yakomaxa/MD_gromacs_template.

## Abbreviations

PDB: Protein data bank
TM-score: Template-match score
ECOD: Evolutionary classification of protein domains
