# Supplementary material for: Enumeration and comprehensive in-silico modeling of three-helix bundle structures composed of typical αα-hairpins
Source: BMC Bioinformatics. 2021 Sep 27;22:465. doi: 10.1186/s12859-021-04380-5 (PMC8474748; doi:10.1186/s12859-021-04380-5)
Supplement: Supplementary file 1 — Additional file 1. Supplementary Figures S1–S45, Supplementary Tables S1 and S2. [file 12859_2021_4380_MOESM1_ESM.pdf]

Supplementary Material for "Enumeration and comprehensive in-silico modeling of three-helix bundle structures composed of typical αα-hairpins"

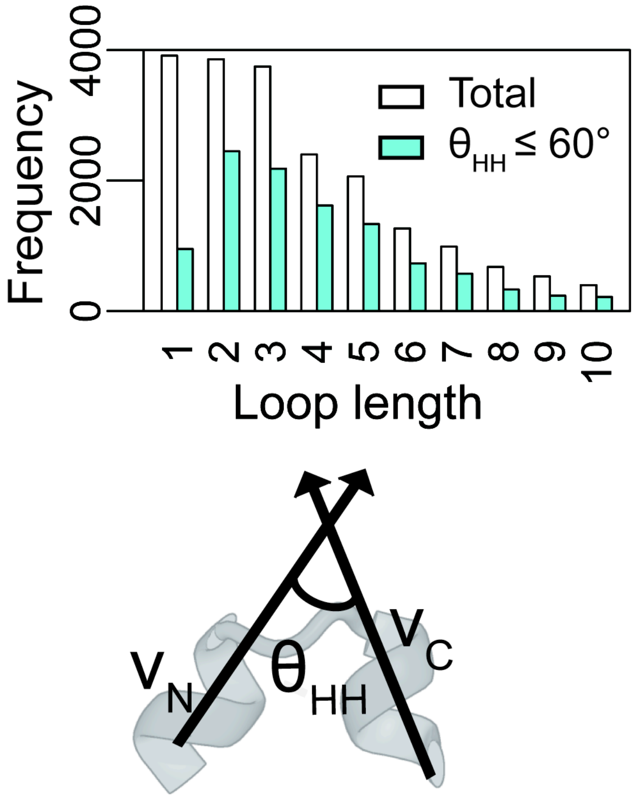

Figure S1. The Distributions of loop lengths. (Top) The distributions of loop lengths in the general helix-loop-helix fragment (white) and αα-hairpins conditioned by θ_{HH} < 60° (cyan). Loop length is the number of residues between two flanking α-helices. (Bottom) The definition of the helix-helix crossing angle θ_{HH}. Shorter loops were generally preferred but the population of single-residue loops diminished when θ_{HH} was less than or equal to 60°. Single-residue loops were too short to form hairpin structures.

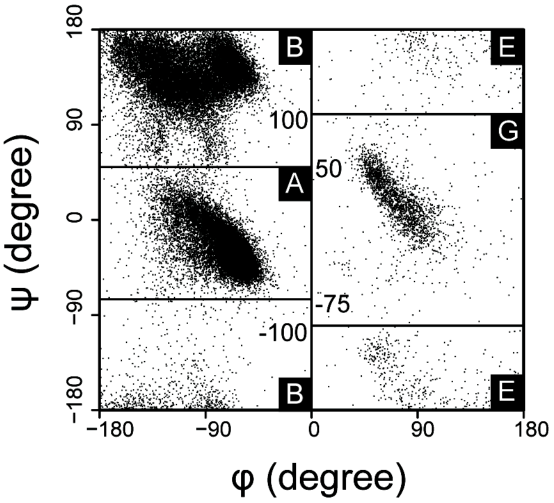

Figure S2. The definition of ABEGO, a coarse-grained backbone torsion representation. ABEGO is a five-state coarse-grained representation of polypeptide backbone dihedral angles; Ramachandran map is divided into four sections and labelled by single letters A, B, E and G, to enable the representation of dihedral angle series by character strings. The A region roughly corresponds to the conformation of α-helix, and the B region corresponds roughly to the β-strand conformation. The G region corresponds to left-handed α-helix, and the E region represents the rest of the Ramachandran map. The O state correspondds to the cis-conformation of peptide bond, which are almost negligible in this paper.

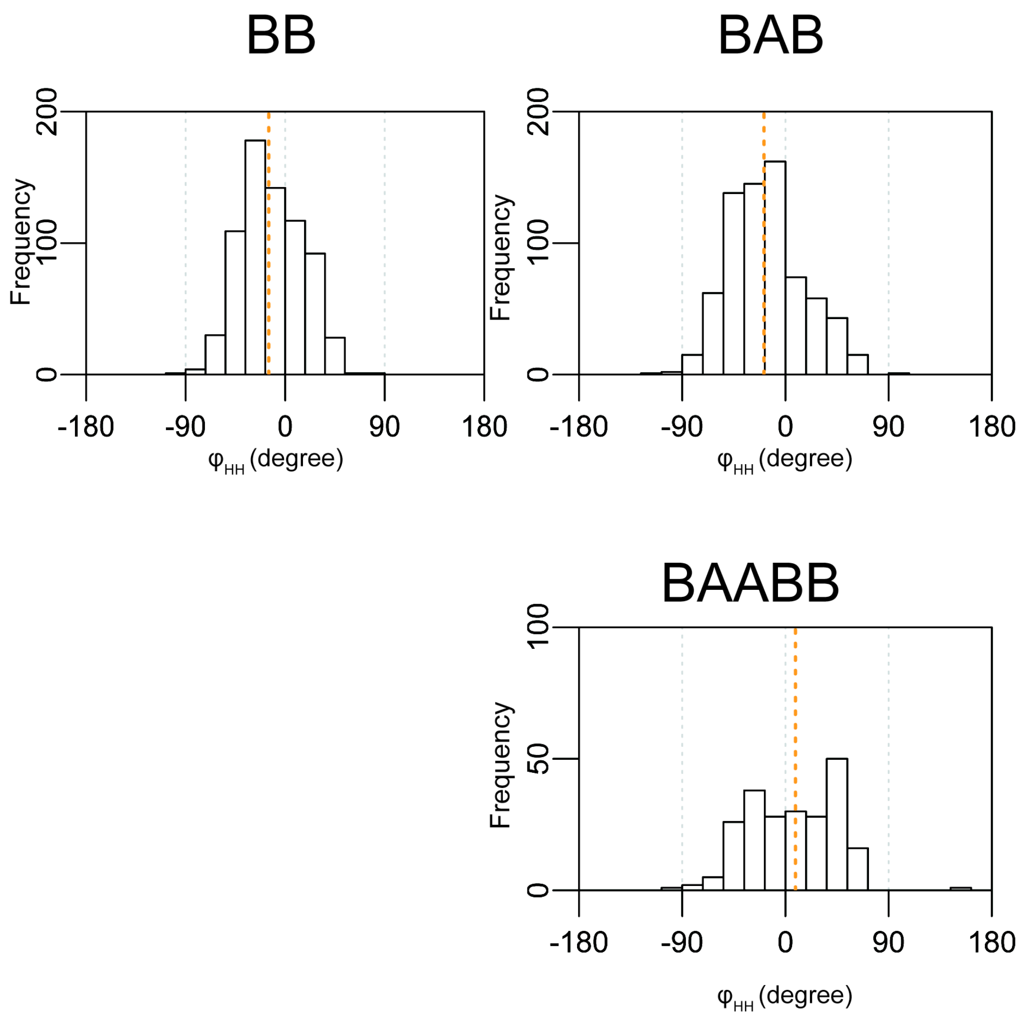

Figure S3. The distribution of φ_{HH} for BB, BAB, and BAABB loops. BB, BAB, and BAABB loops exhibited undetermined distributions of φ_{HH}, showing broad spectra around φ_{HH} = 0. The orange dotted line indicates the median of the distributions. These show that these BB, BAB, and BAABB hairpins cannot specify the handedness of the helix-helix packing, which we omitted in the later analysis.

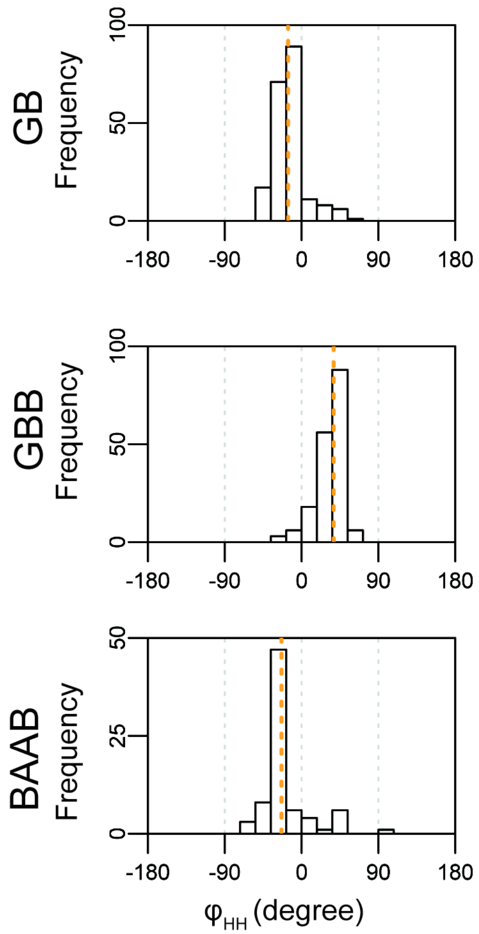

Figure S4: The distribution of φ_{HH} for GB, GBB, and BAAB loops using high-resolution structure dataset.

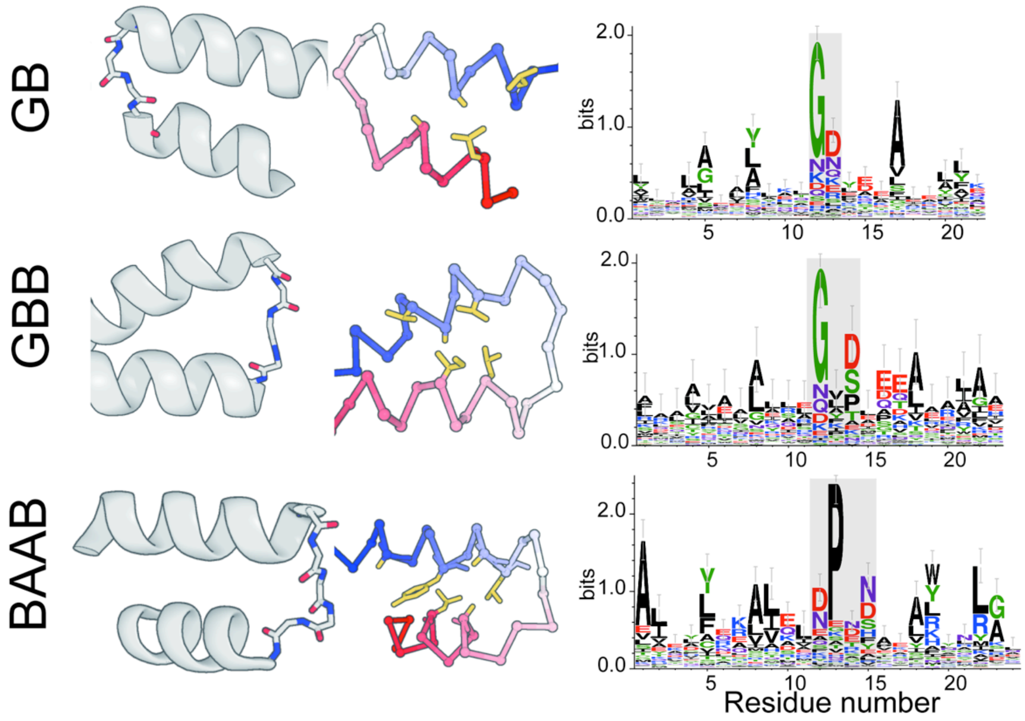

Figure S5. Packing between two α-helices connected by typical hairpins. (Left) The representative structures of typical hairpins. The α-helices are represented as cartoons, and loop regions are shown as sticks. (Middle) Hydrophobic residues enable tight packing between two α-helices in the representative structures. The hydrophobic residues are shown as orange sticks, and the backbones are shown as Cα-traces. (Left) The structure-based sequence alignments are shown as the sequence logo for the hairpin and the flanking ten residues of α-helices. The gray-shaded boxes indicate hairpin loop regions. There are hallmarks of hydrophobic helix-helix packing motifs in the α-helix regions, where small and hydrophobic residues periodically appear in the sequences for tight Van-der-Waals contacts.

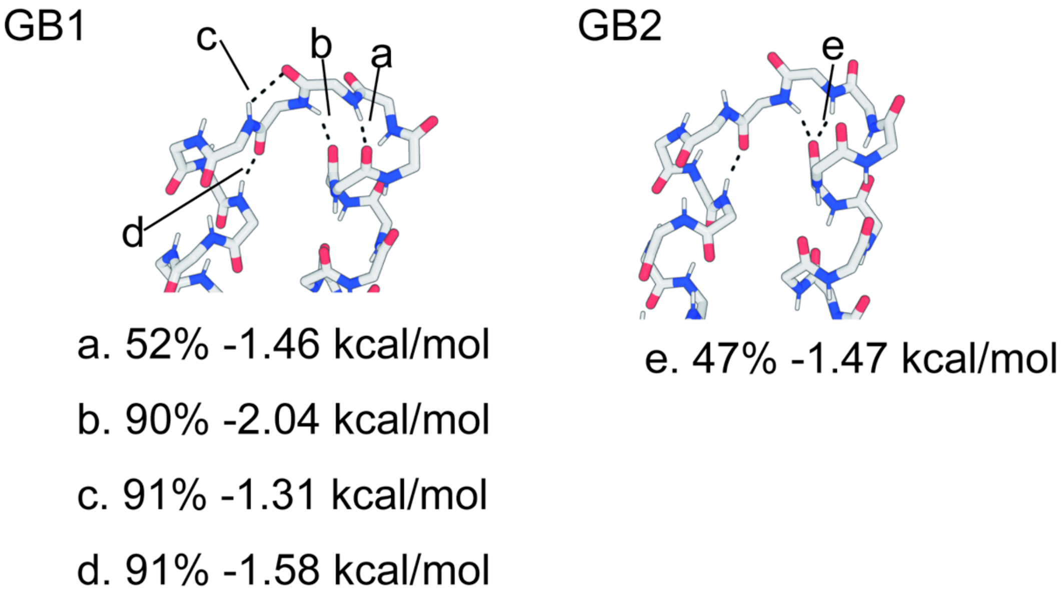

Figure S6: Hydrogen bond patterns in GB-loop. The percentage indicates the ratio of hydrogen bond formation in the dataset. The energy values represent the average bonding energy for each hydrogen bond estimated by DSSP. The GB-loop typically has 3 or 4 intra-loop hydrogen bonds that stabilize the loop conformation.

GBB

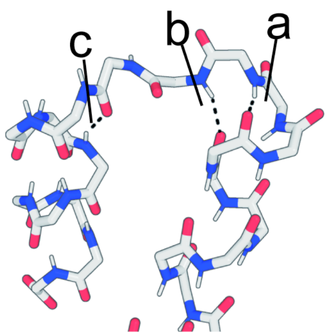

a. 87% -1.00 kcal/mol

b. 85% -1.99 kcal/mol

c. 96% -2.31 kcal/mol

Figure S7: Hydrogen bond patterns in GBB-loop. The percentage indicates the ratio of hydrogen bond formation in the dataset. The energy values represent the average bonding energy for each hydrogen bond estimated by DSSP. The GBB-loop typically has 3 intra-loop hydrogen bonds that stabilize the loop conformation.

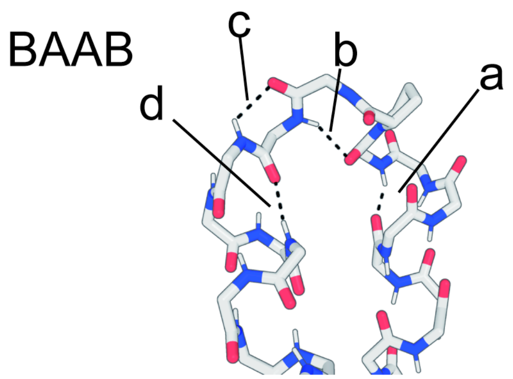

a. 94% -2.21 kcal/mol

b. 98% -1.61 kcal/mol

c. 87% -1.00 kcal/mol

d. 82% -1.66 kcal/mol

Figure S8: Hydrogen bond patterns in BAAB-loop. The percentage indicates the ratio of hydrogen bond formation in the dataset. The mean energy values represent the average bonding energy for each hydrogen bond estimated by DSSP. The GBB-loop typically has 4 intra-loop hydrogen bonds that stabilize the loop conformation.

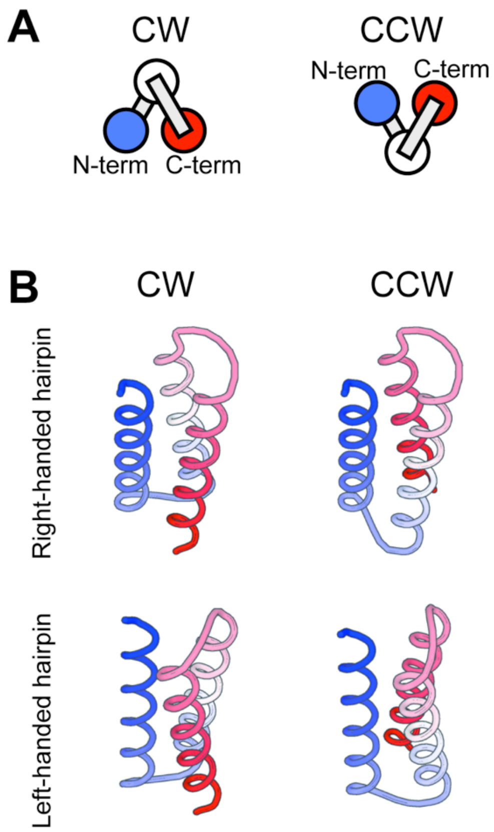

Figure S9. Two possible chiral forms of three-helix bundles and decoy structures used in the evaluation of sequence-independent folding simulations. (A) Three-helical bundles can have two types of chirality in their overall structures: Clockwise (CW) and Counterclockwise (CCW). Please note that this is independent of the local hairpin motifs. Circles indicate the α-helix viewed from the top, and bars indicate the connecting loops. (B) There are four possible three-helical bundle structures when local handedness of αα-hairpins are also considered. We used the right-handed decoys for GBB-bundle folding simulations, and the left-handed decoys for the GB and BAAB-bundle simulations.

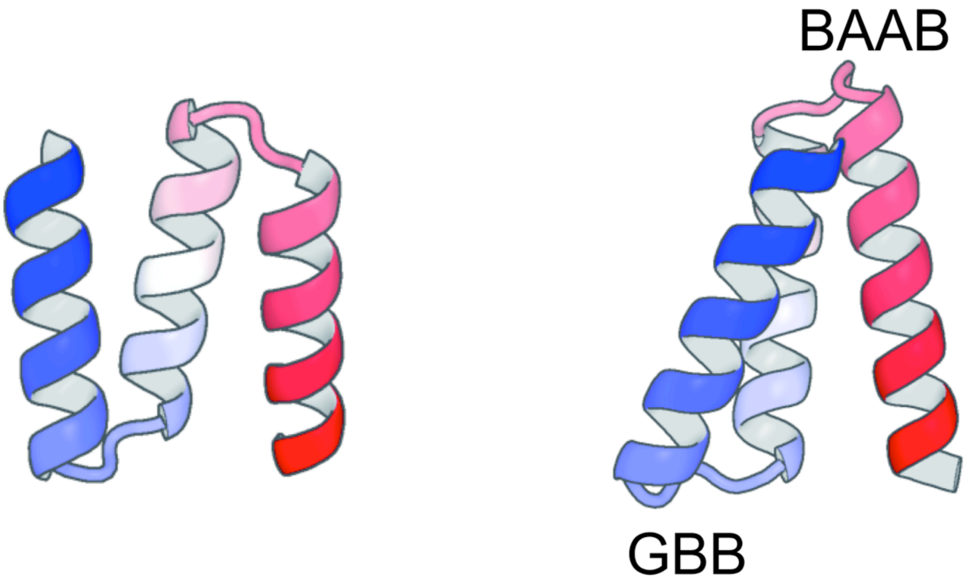

Figure S10. Two examples of poorly packed three-helical bundle structures. (Left) When two GB-hairpins are connected by a15 residue helix in the middle, the first and third helices are placed apart and result in an extended confirmation lacking contacts between the first and third helix. This type of extended structure lacks the would-be-hydrophobic-core region and therefore is not considered designable. (Right) An example of the structure with left- and right-handed mixed hairpins. The first and third α-helix can not pack when the hairpins with different handedness are mixed, and do not yield compact and globular three-helical bundle structures. See also Figure S15-S18, where such mixed-loop simulations are shown to be unable to generate either CW or CCW compact bundle structures.

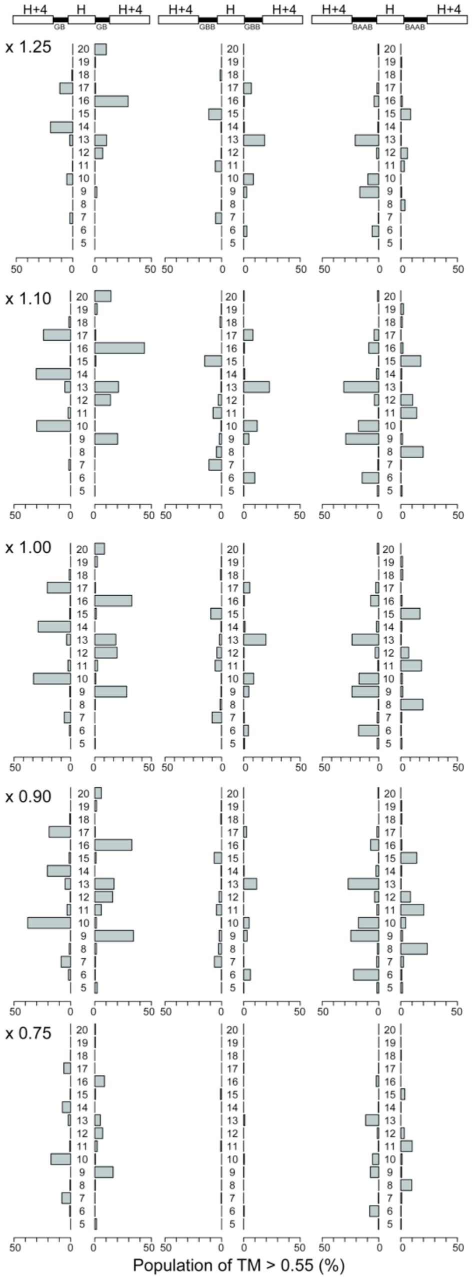

Figure S11: Effect of reference structures for TM-score calculations in the analysis on backbone-building simulations. The value at the upper left of each panel represents the magnification factor for the diameter of reference helix-bundles.This figure corresponds to figure 2 in main text, and shows that the results are not severely affected by the change in reference structures for TM-score calculation.

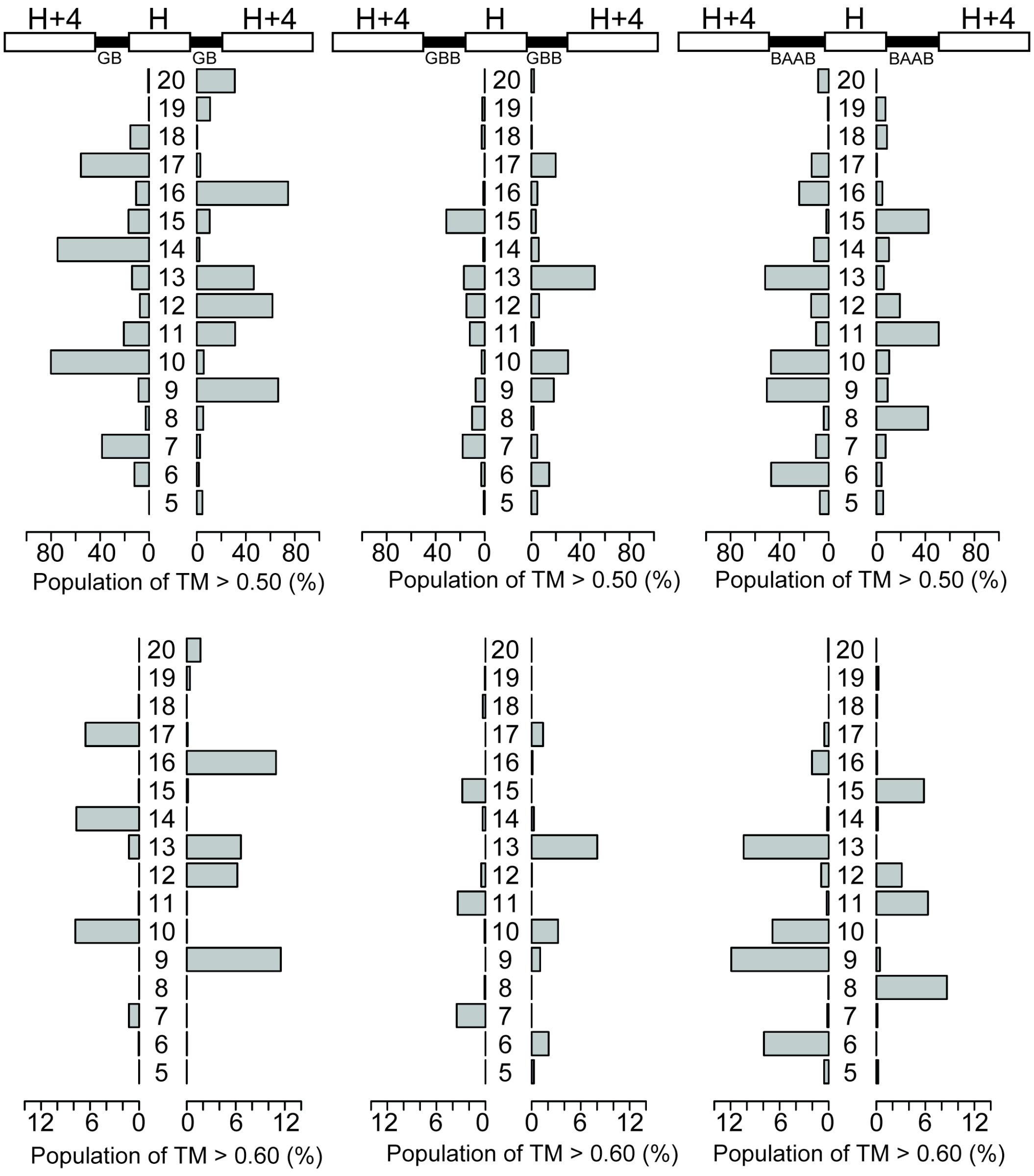

Figure S12: Effect of cut-off for TM-score in the analysis on backbone-building simulations. Two different thresholds 0.50 and 0.60 are used instead of the original threshold 0.55. This figure corresponds to figure 2 in the main text, and shows that the results are not severely affected by the change in TM-score threshold..

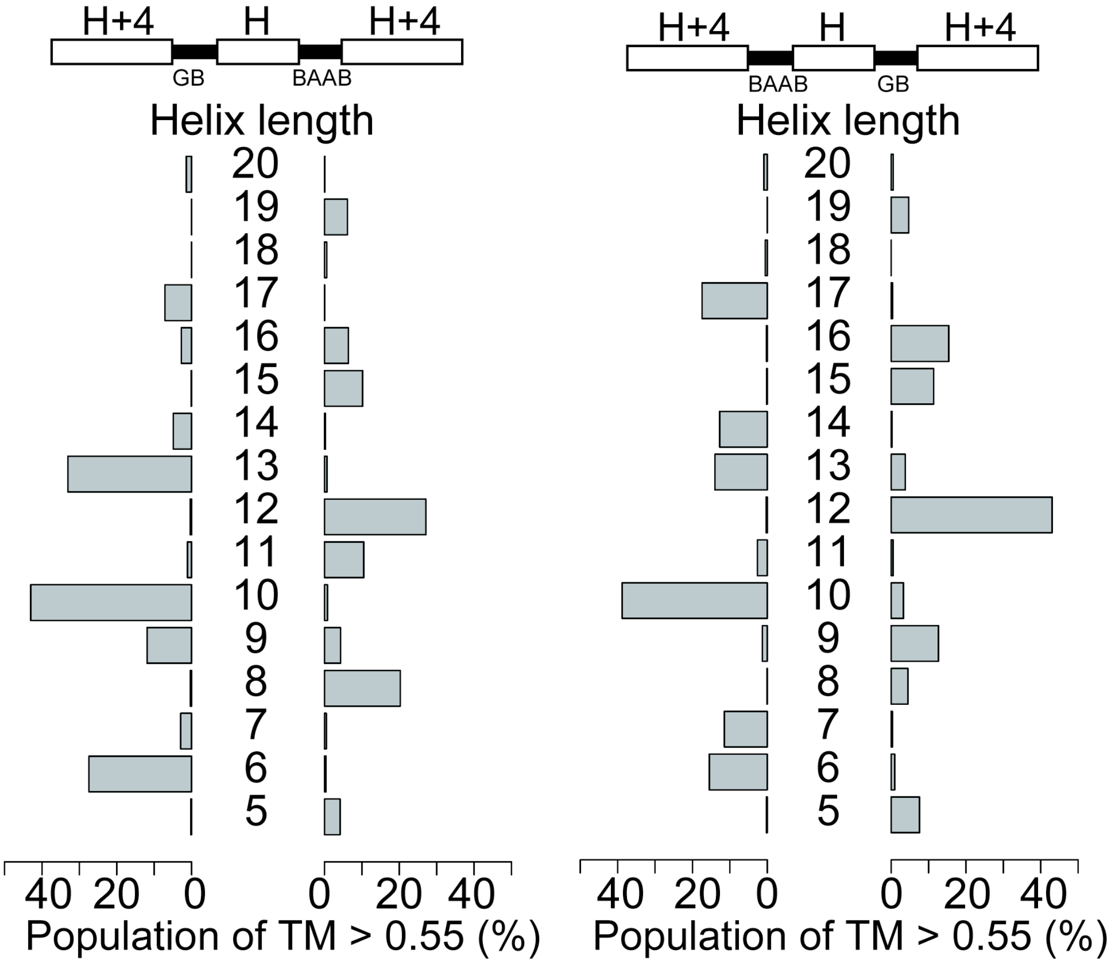

Figure S13. The blueprints and result of backbone-building simulations with blueprints containing two different loop types. (left) the GB-BAAB blueprint (Right) the BAAB-GB blueprints. As the GB and BAAB hairpins are both left-handed type hairpins, the helix-helix crossing angle cancels out so that the mixture of the GB and BAAB-loops can yield compact three-helix bundles in similar manners as GB-GB or BAAB-BAAB blueprints.

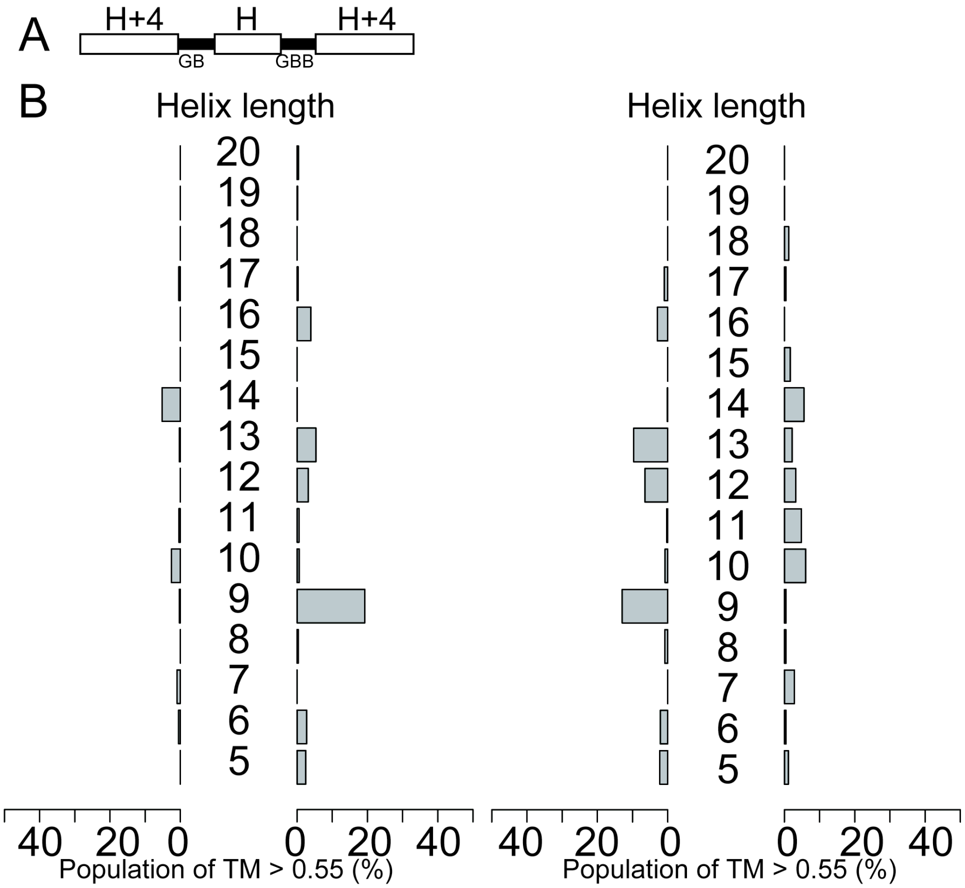

Figure S14. Backbone-building simulations with blueprints containing two different loop types. (A) The GB-GBB blueprint (B) Results of GB-GBB blueprint simulation referenced by CW decoy (left) GB-GBB blueprint simulation referenced by CCW decoy(right). Almost no patterns can be observed compared to the consistent blueprint such as GB-GB blueprints because combination of GB and GBB cannot cancel out the helix-helix packing angles.

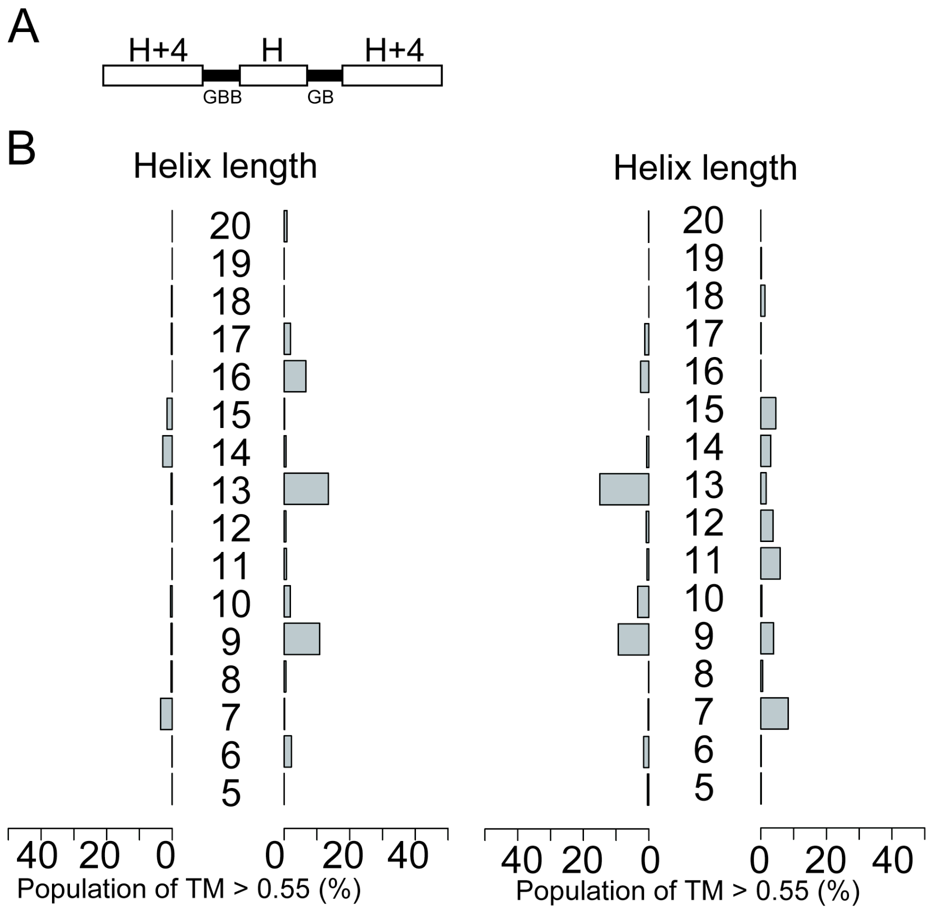

Figure S15. Backbone-building simulations with blueprints containing two different loop types. (A) The GBB-GB blueprint (B) Results of GBB-GB blueprint simulations referenced by CW decoy (left) GBB-GB blueprint simulations referenced by CCW decoy (right). Almost no patterns can be observed compared to the consistent blueprint such as GB-BAAB blueprint because combination of GB and GBB cannot cancel out the helix-helix packing angles

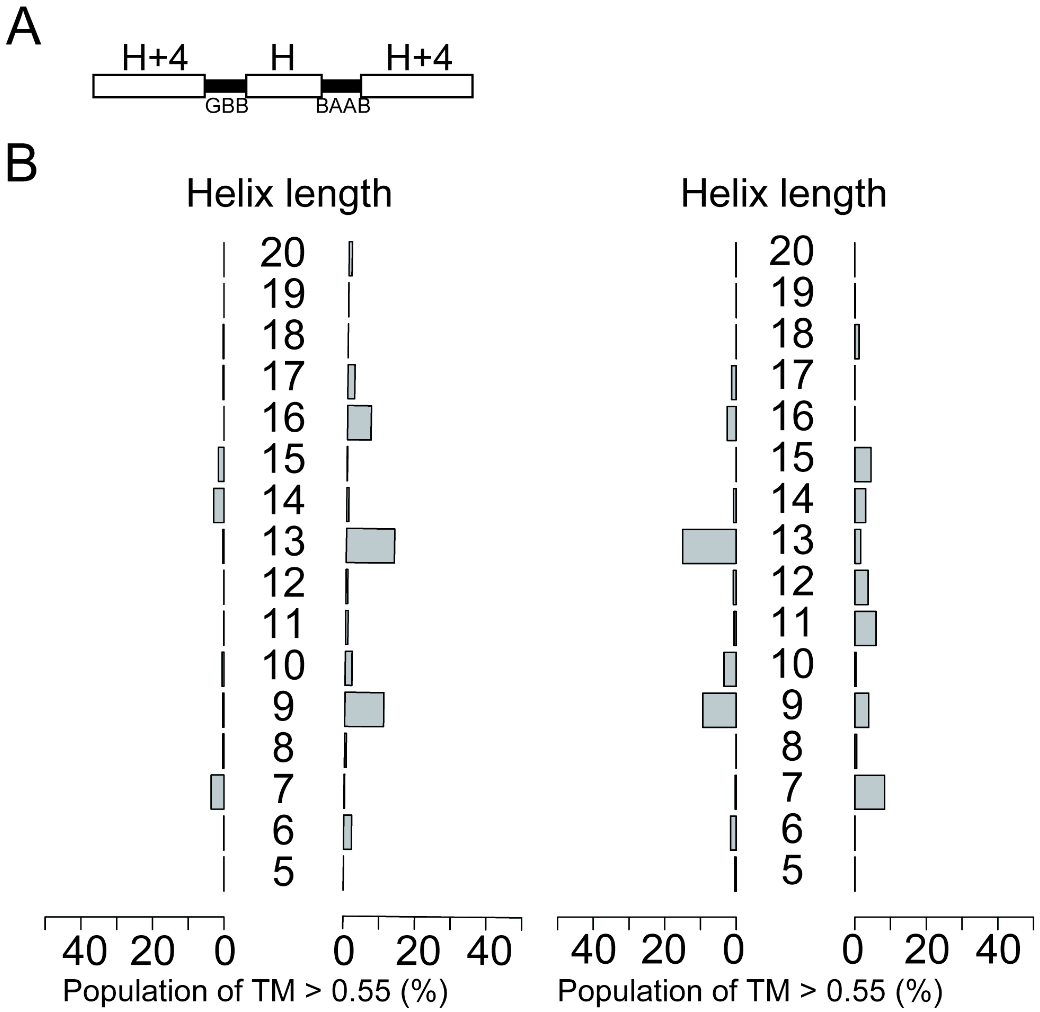

Figure S16. Backbone-building simulations with blueprints containing two different loop types. (A) The GBB-BAAB blueprint (B) Results of GBB-BAAB blueprint simulations referenced by CW decoy (left) GB-GBB blueprint simulation referenced by CCW decoy (right). Almost no patterns can be observed compared to the consistent blueprint such as GB-GB blueprints because combination of BAAB and GBB cannot cancel out the helix-helix packing angles.

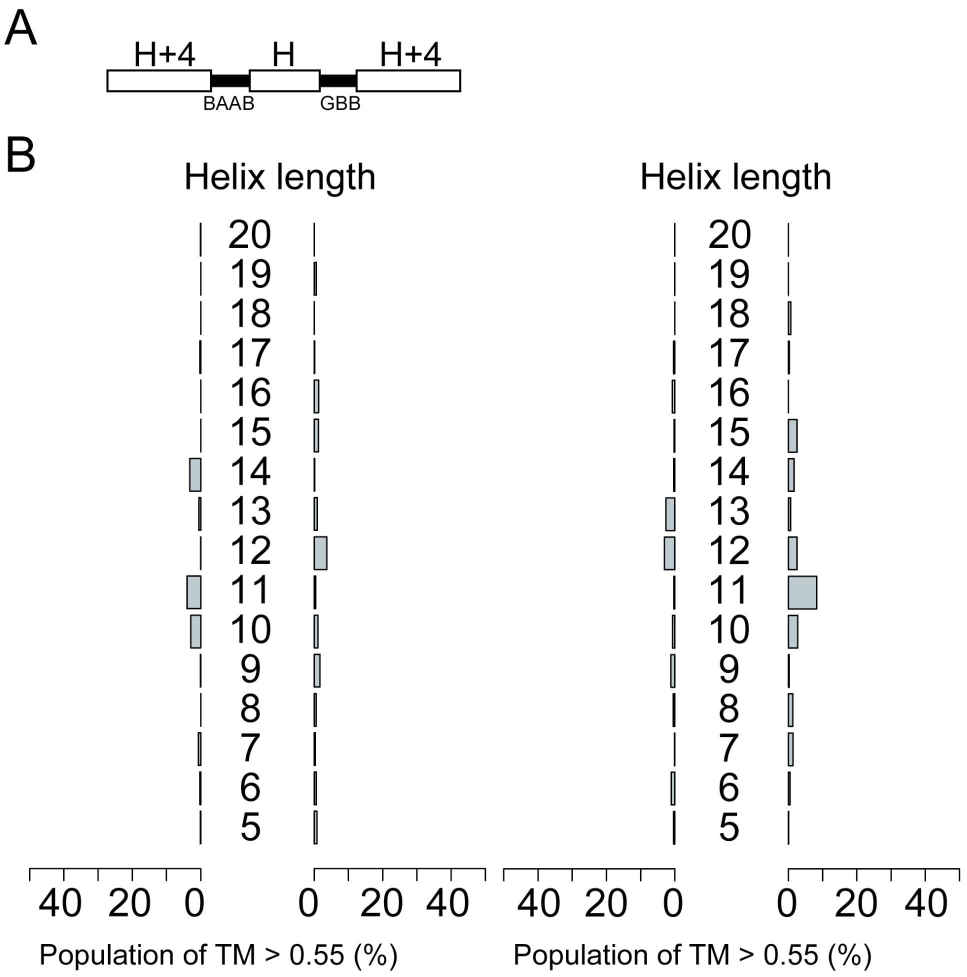

Figure S17. Backbone-building simulations with blueprints containing two different loop types. (A) The BAAB-GBB blueprint (B) Results of BAAB-GBB blueprint simulation referenced by CW decoy (left) BAAB-GBB blueprint simulation referenced by CCW decoy (right). Almost no patterns can be observed compared to the consistent blueprint such as GB-GB blueprints because combination of BAAB and GBB cannot cancel out the helix-helix packing angles.

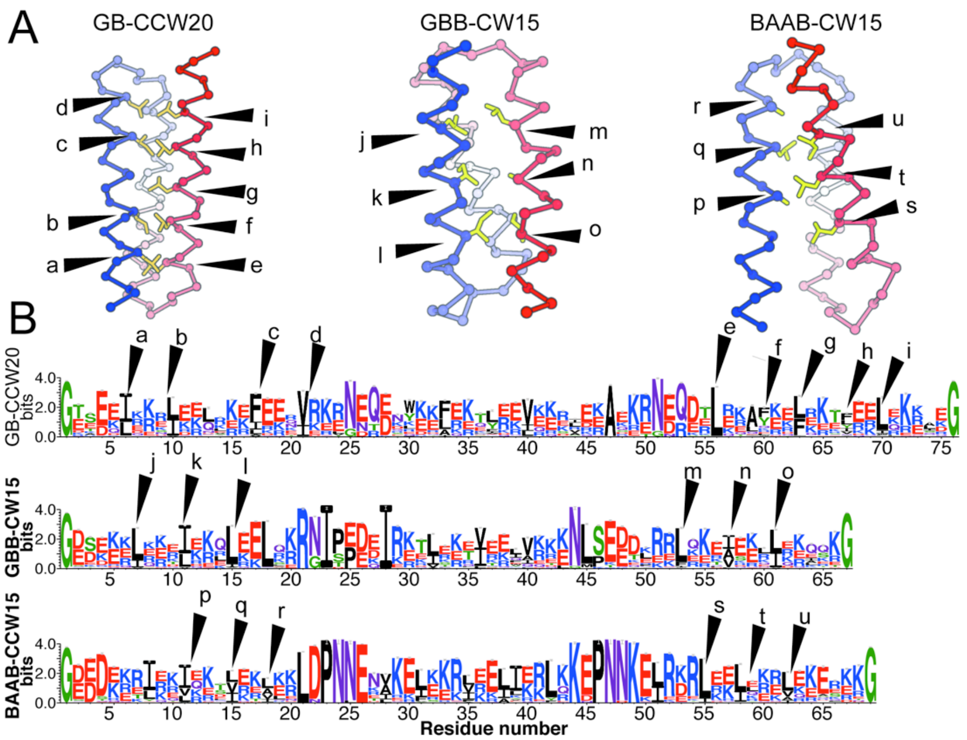

Figure S18. The side-chain packing in the interface between first and third α-helices in the structures of designed three-helix bundles, GB-CCW20, GBB-CW15, and BAAB-CCW15. (A) The structures of representative three-helix bundles designed. The chains are colored in blue-white-red gradient from N-term to C-term, where the first/third α-helix is colored approximately in blue/red. The side-chain atoms are represented as yellow sticks, and Cα atoms are represented as spheres. (B) The sequence profiles of all of the designed sequences aligned. The alphabets indicate which residue in the structures corresponds to the site in the profiles. Hydrophobic residues appear in every three or four residues and form tight packing between first and third α-helices.

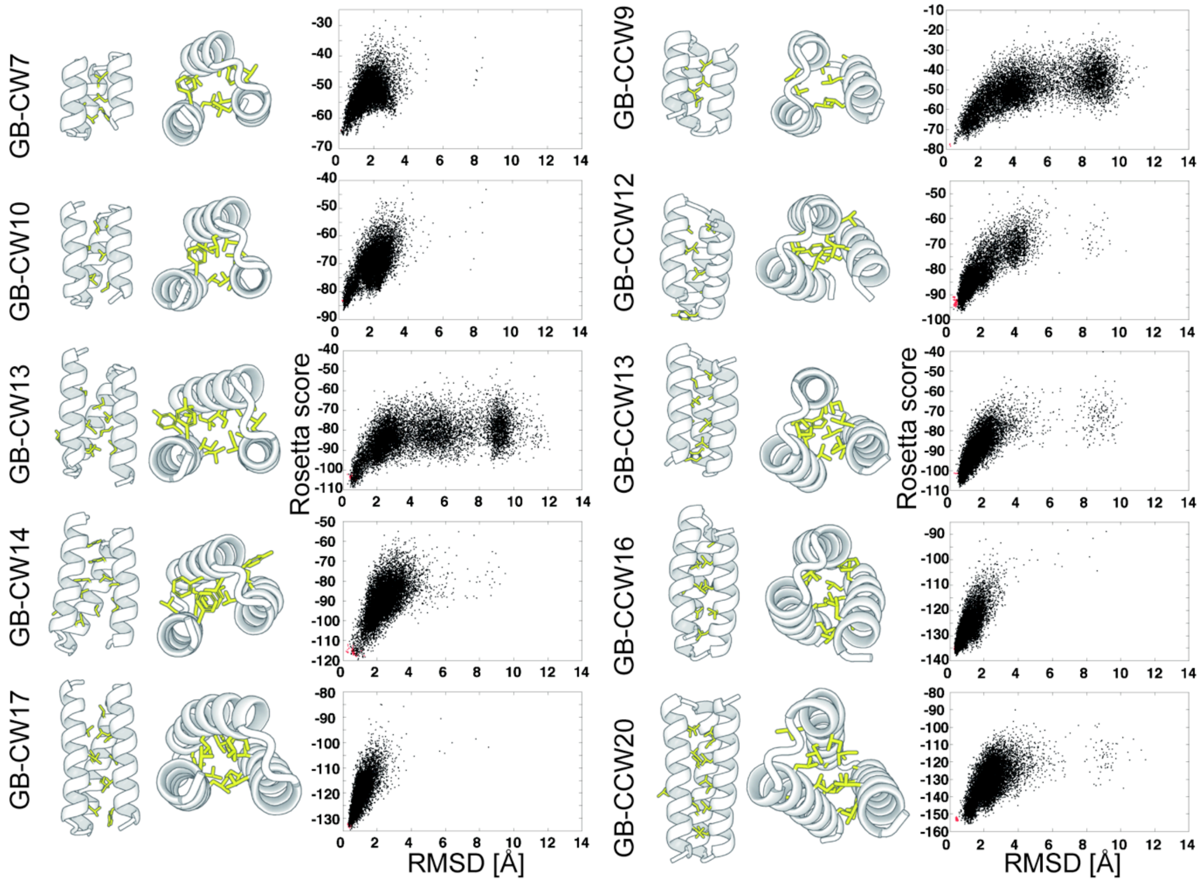

Figure S19. Structures and folding-funnels of GB-bundles. (Left) The side-view of the designed structures with α-helix shown as cartoon and hydrophobic side-chains represented as sticks. (Center) The top-view of the designed structures. (Right) The result of folding simulations. The vertical axis represents the Rosetta score, and the horizontal axis represents the RMSD from the target structures. The black dots correspond to the final snapshots of the fragment-assembly folding simulations starting from extended conformations, and red dots correspond to the final snapshots of relax-simulation starting from native conformations.

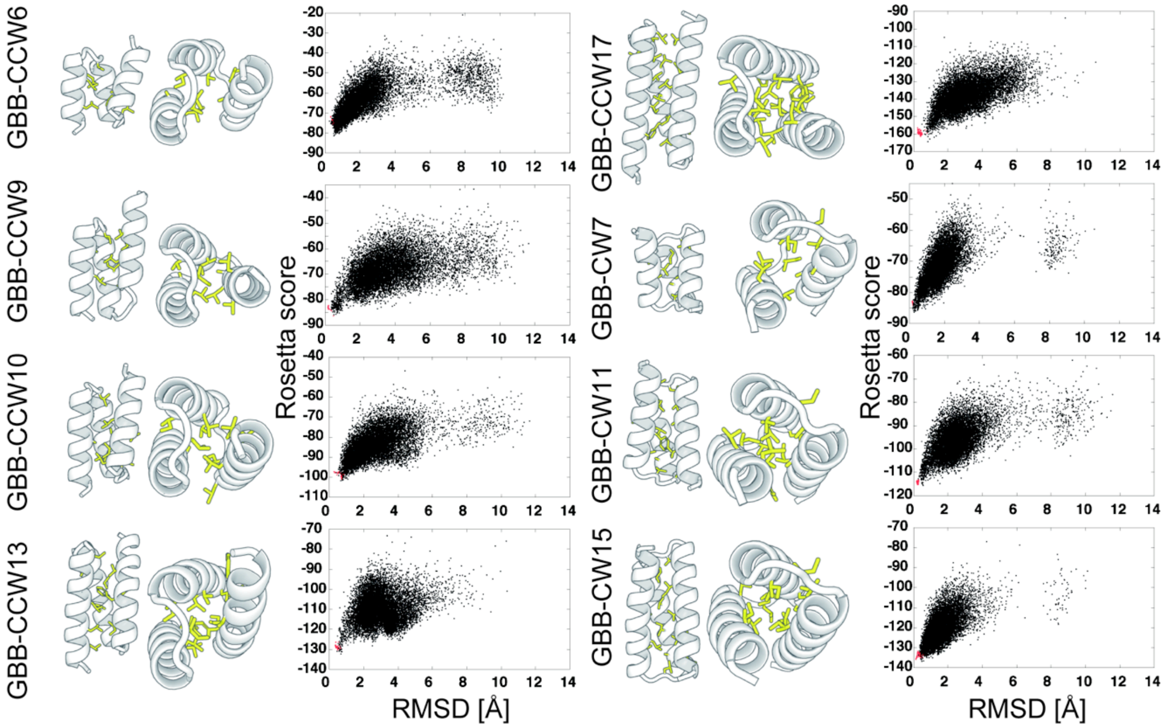

Figure S20. Structures and folding-funnels of GBB-bundles. (Left) The side-view of the designed structures with α-helix shown as cartoon and hydrophobic side-chains represented as sticks. (Center) The top-view of the designed structures. (Right) The result of folding simulations. The vertical axis represents the Rosetta score, and the horizontal axis represents the RMSD from the target structures. The black dots correspond to the final snapshots of the fragment-assembly folding simulations starting from extended conformations, and red dots correspond to the final snapshots of relax-simulation starting from native conformations.

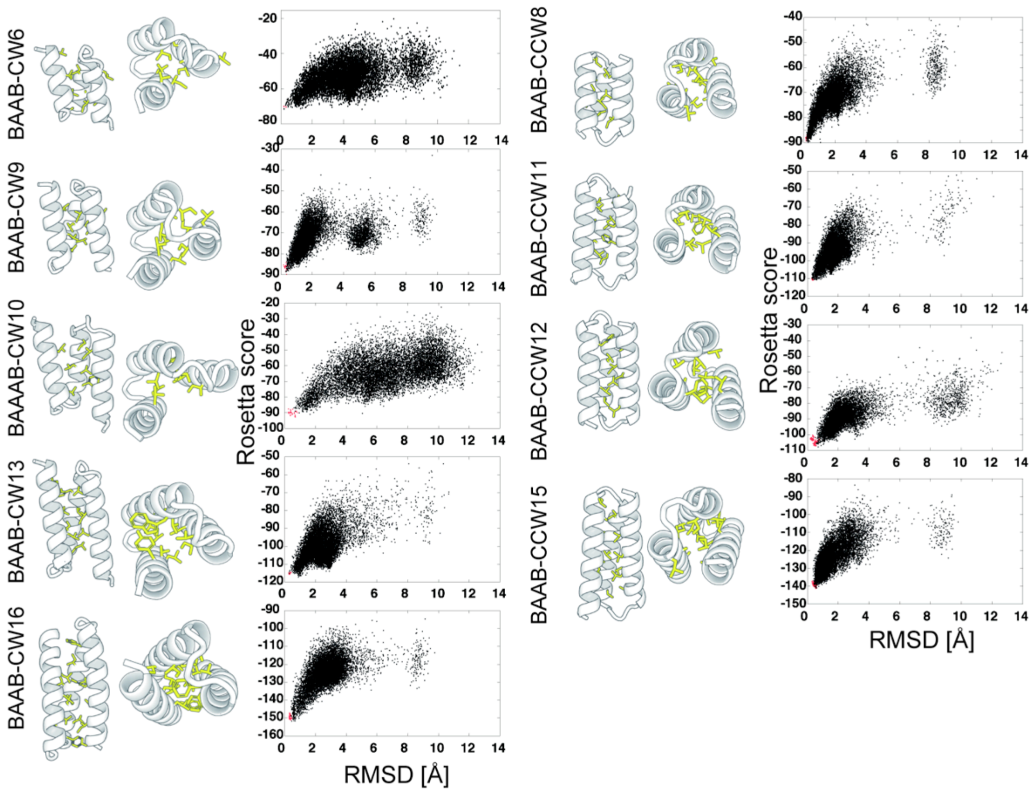

Figure S21. Structures and folding-funnels of BAAB-bundles. (Left) The side-view of the designed structures with α-helix shown as cartoon and hydrophobic side-chains represented as sticks. (Center) The top-view of the designed structures. (Right) The result of folding simulations. The vertical axis represents the Rosetta score, and the horizontal axis represents the RMSD from the target structures. The black dots correspond to the final snapshots of the fragment-assembly folding simulations starting from extended conformations, and red dots correspond to the final snapshots of relax-simulation starting from native conformations.

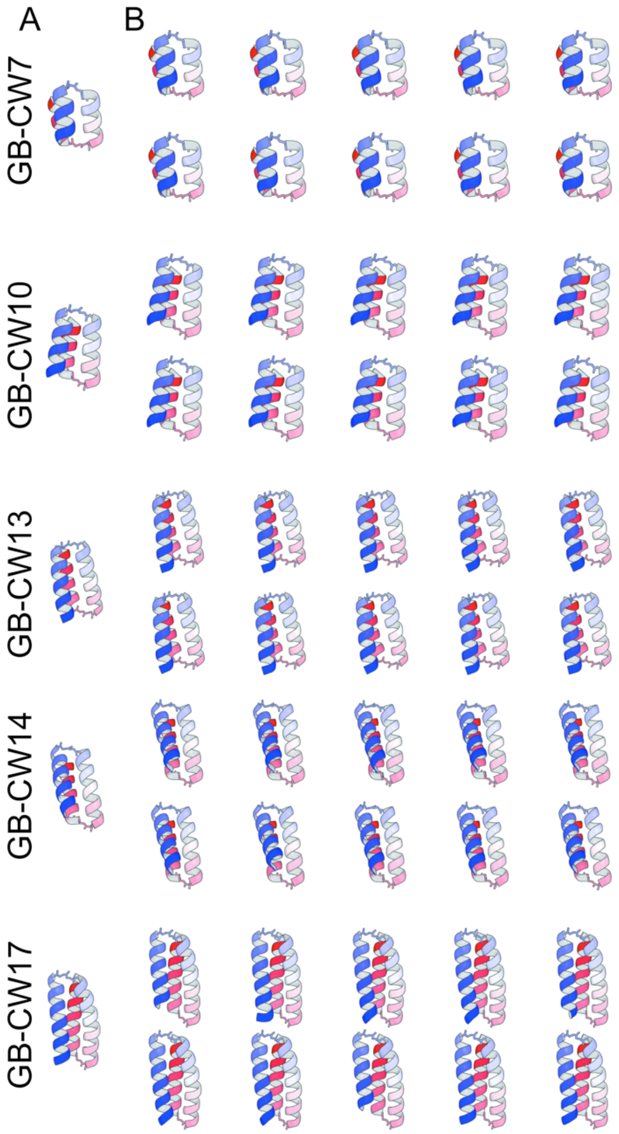

Figure S22. Comparison of the design structures composed of the GB-hairpins (A) and the 10 lowest score predictions by sequence-dependent folding simulations (B). Loops are shown as sticks in order to show the detailed conformations. The predictions precisely recovered the local conformations in most of the lowest score models. Overall topologies of predicted models agree with design models.

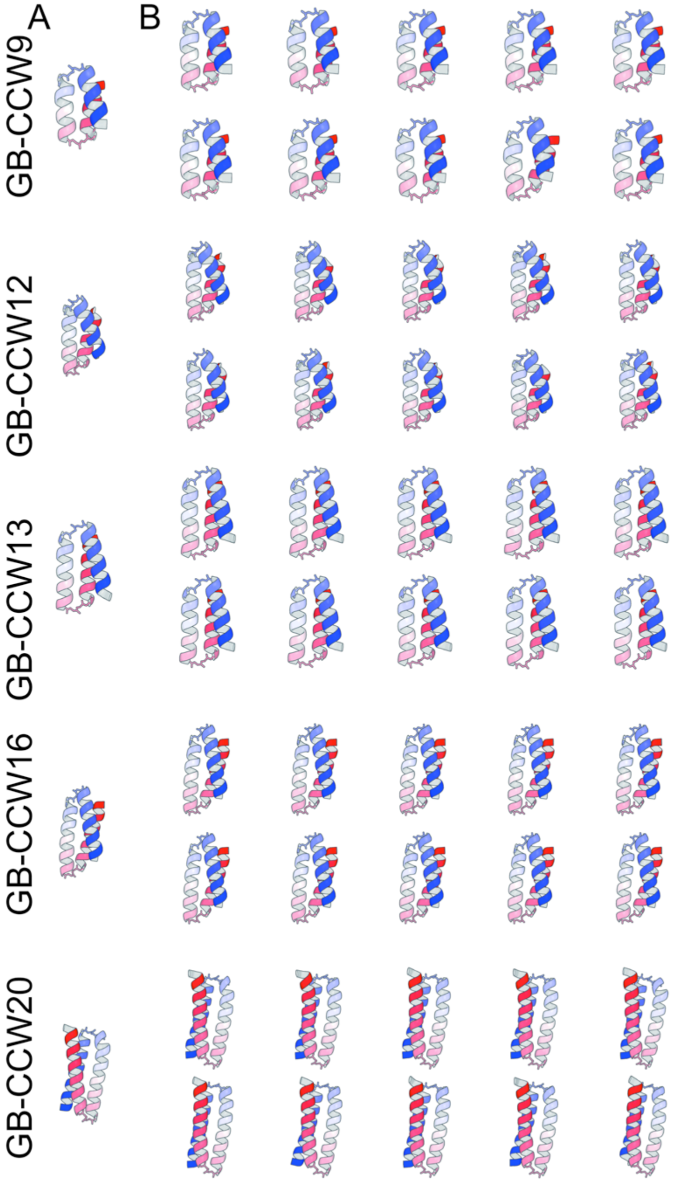

Figure S23. Comparison of the design structures composed of the GB-hairpins (A) and the 10 lowest score predictions by sequence-dependent folding simulations (B). Loops are shown as sticks in order to show the detailed conformations. The predictions precisely recovered the local conformations in most of the lowest score models. Overall topologies of predicted models agree with design models.

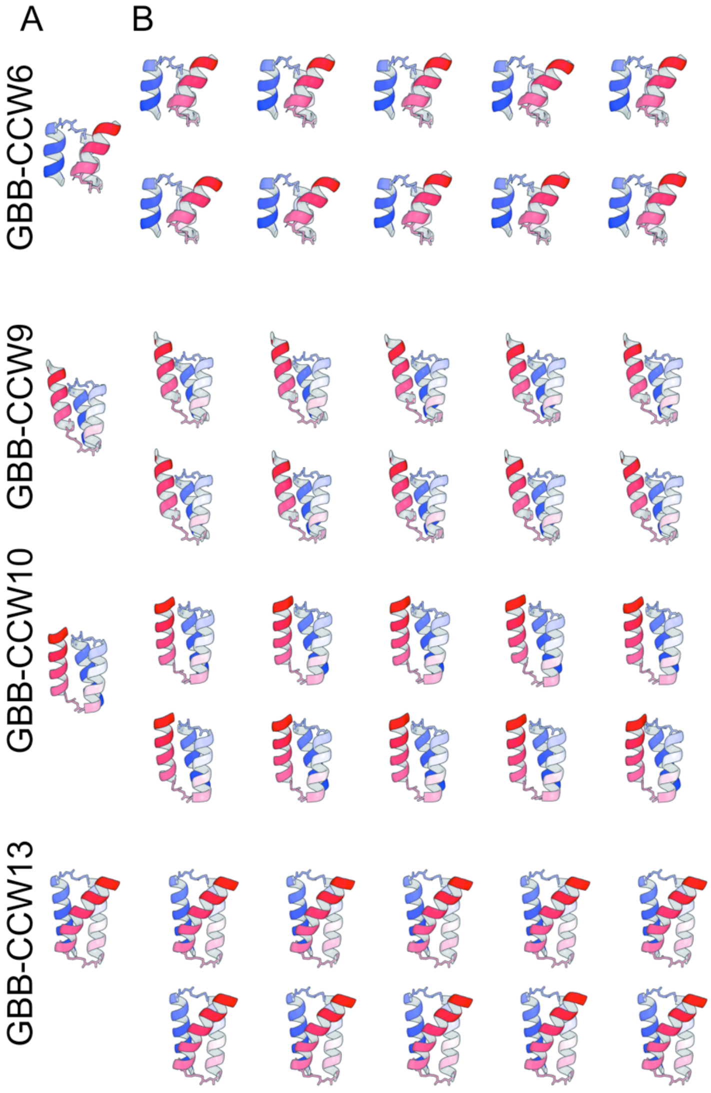

Figure S24. Comparison of the design structures composed of the GBB-hairpins (A) and the 10 lowest score predictions by sequence-dependent folding simulations (B). Loops are shown as sticks in order to show the detailed conformations. The predictions precisely recovered the local conformations in most of the lowest score models. Overall topologies of predicted models agree with design models.

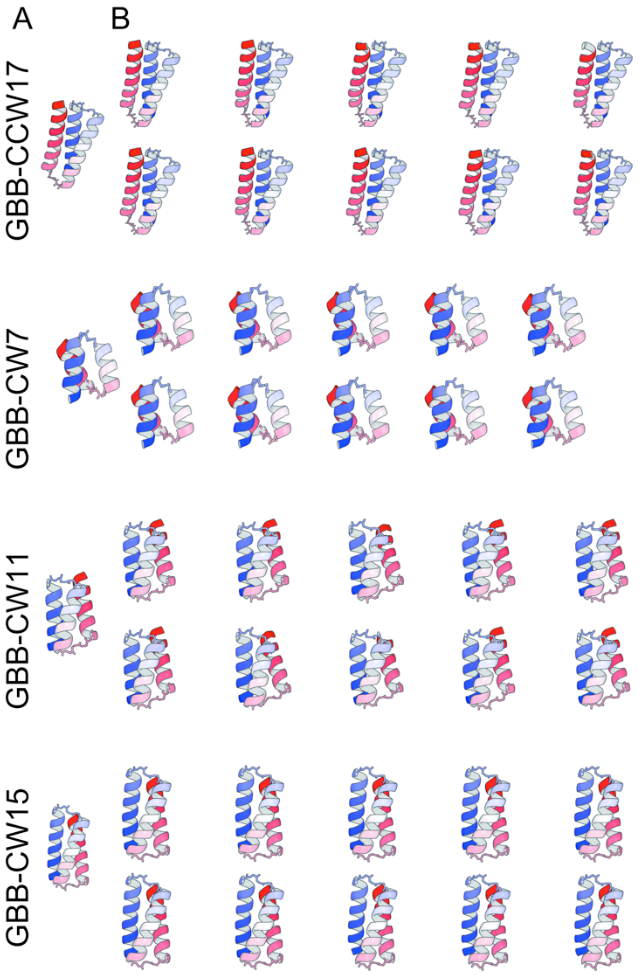

Figure S25. Comparison of the design structures composed of the GBB-hairpins (A) and the 10 lowest score predictions by sequence-dependent folding simulations (B). Loops are shown as sticks in order to show the detailed conformations. The predictions precisely recovered the local conformations in most of the lowest score models. Overall topologies of predicted models agree with design models.

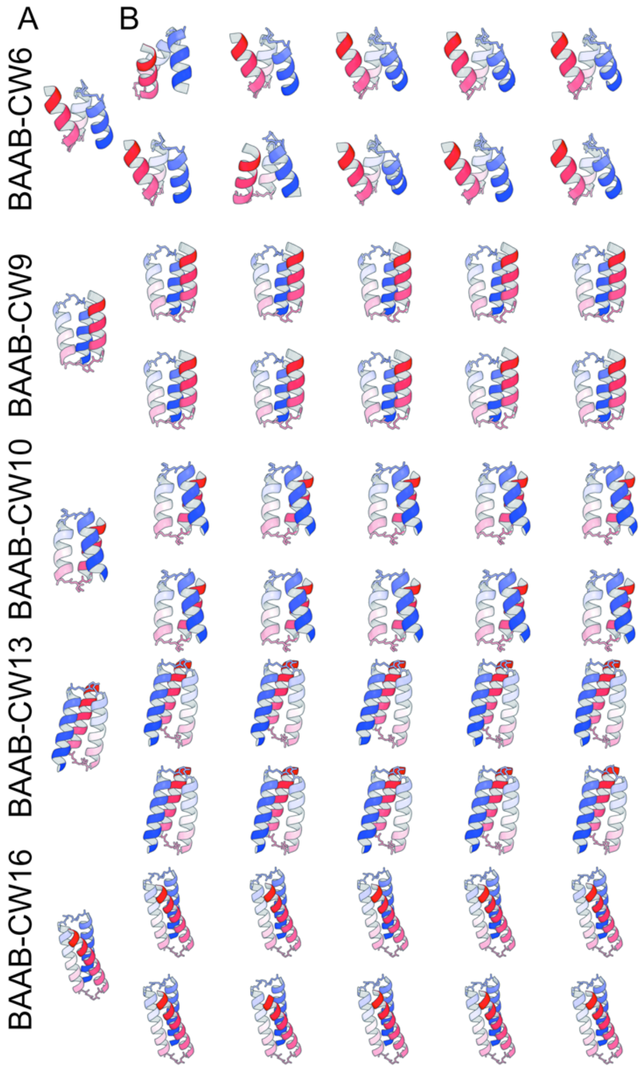

Figure S26. Comparison of the design structures composed of the BAAB-hairpins (A) and the 10 lowest score predictions by sequence-dependent folding simulations (B). Loops are shown as sticks in order to show the detailed conformations. The predictions precisely recovered the local conformations in most of the lowest score models. Overall topologies of predicted models agree with design models.

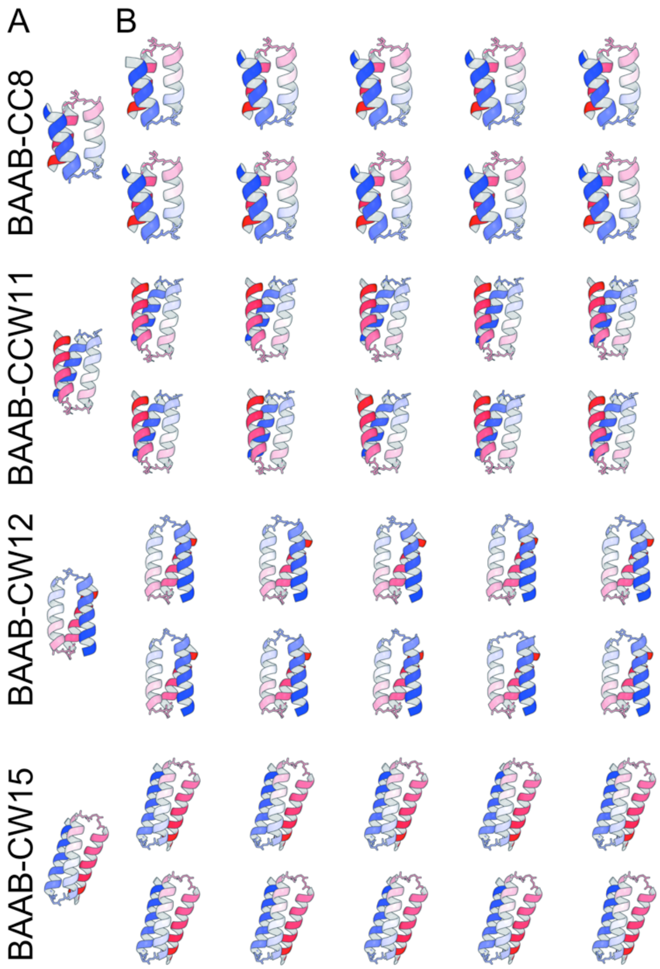

Figure S27. Comparison of the design structures composed of the BAAB-hairpins (A) and the 10 lowest score predictions by sequence-dependent folding simulations (B). Loops are shown as sticks in order to show the detailed conformations. The predictions precisely recovered the local conformations in most of the lowest score models. Overall topologies of predicted models agree with design models.

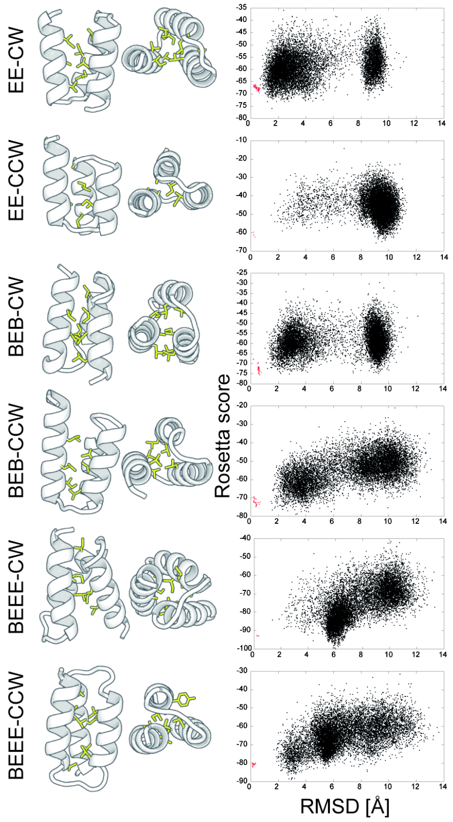

Figure S28: Structures and folding-funnels of best-effort-design three-helix bundle composed of atypical hairpin structures. (Left) The side-view of the designed structures with α-helix shown as cartoon and hydrophobic side-chains represented as sticks. (Center) The top-view of the designed structures. (Right) The result of folding simulations. The vertical axis represents the Rosetta score, and the horizontal axis represents the RMSD from the target structures. The black dots correspond to the final snapshots of the fragment-assembly folding simulations starting from extended conformations, and red dots correspond to the final snapshots of relax-simulation starting from native conformations. These folding-funnels are flat-bottomed and unable to reach the near-native structures.

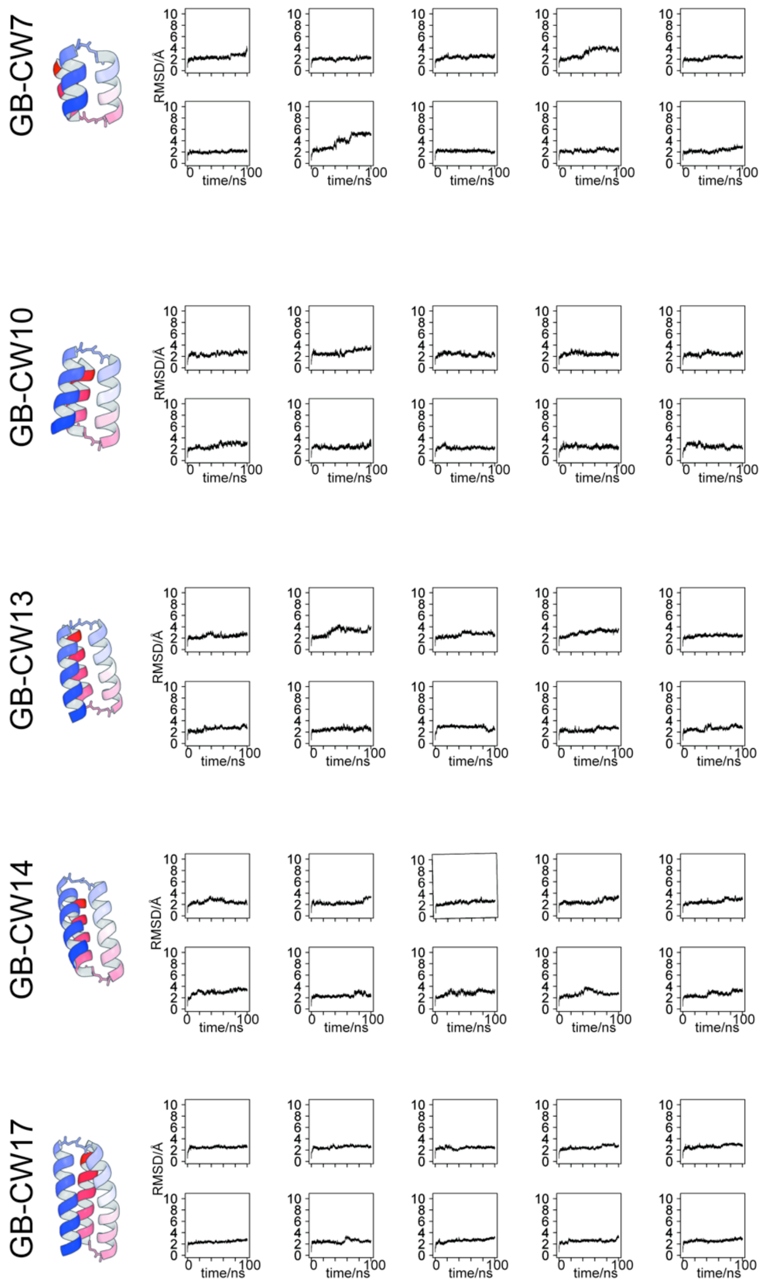

Figure S29: Time series of RMSD in 10 trajectories of 100 ns molecular dynamics simulations. (Left) Structure of designed proteins (Right) Time series of Cα RMSD referenced by the designed protein structure. The horizontal axis represents times in ns, and the veritcal axis represents RMSD in Å.

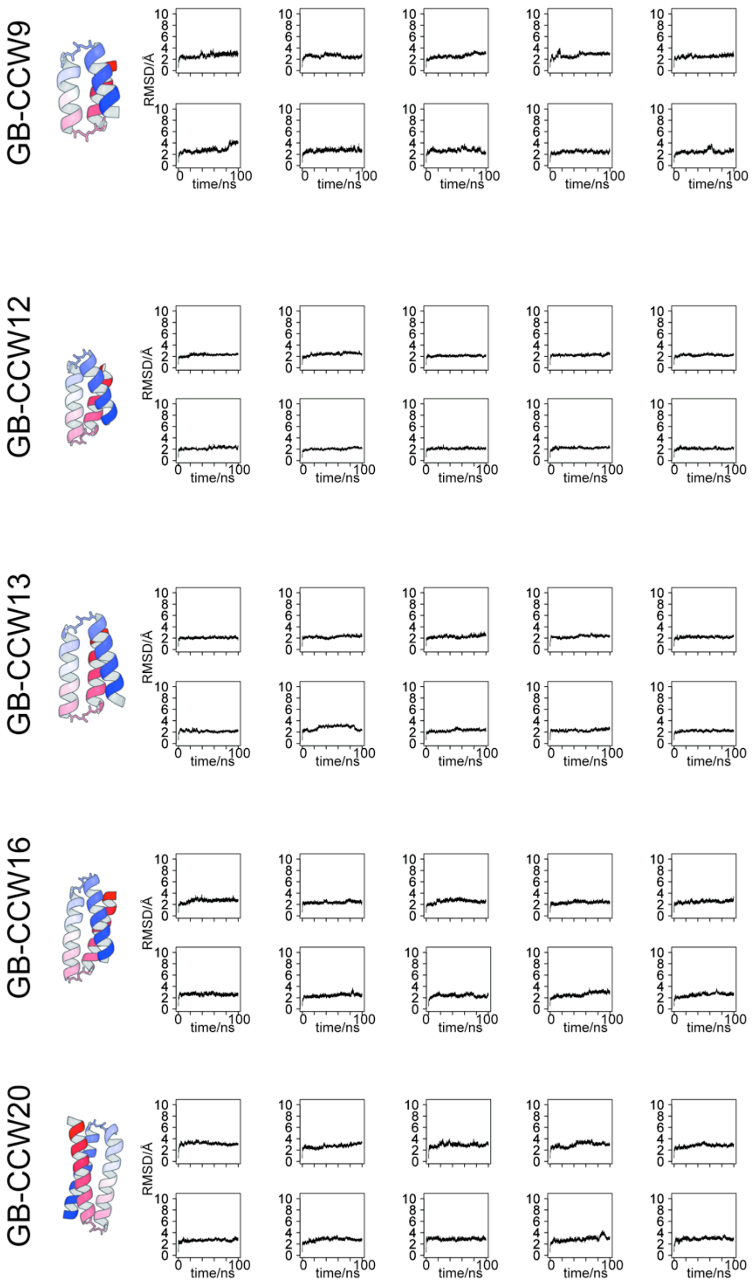

Figure S30: Time series of RMSD in 10 trajectories of 100 ns molecular dynamics simulations. (Left) Structure of designed proteins (Right) Time series of Cα RMSD referenced by the designed protein structure. The horizontal axis represents times in ns,

and the veritcal axis represents RMSD in Å.

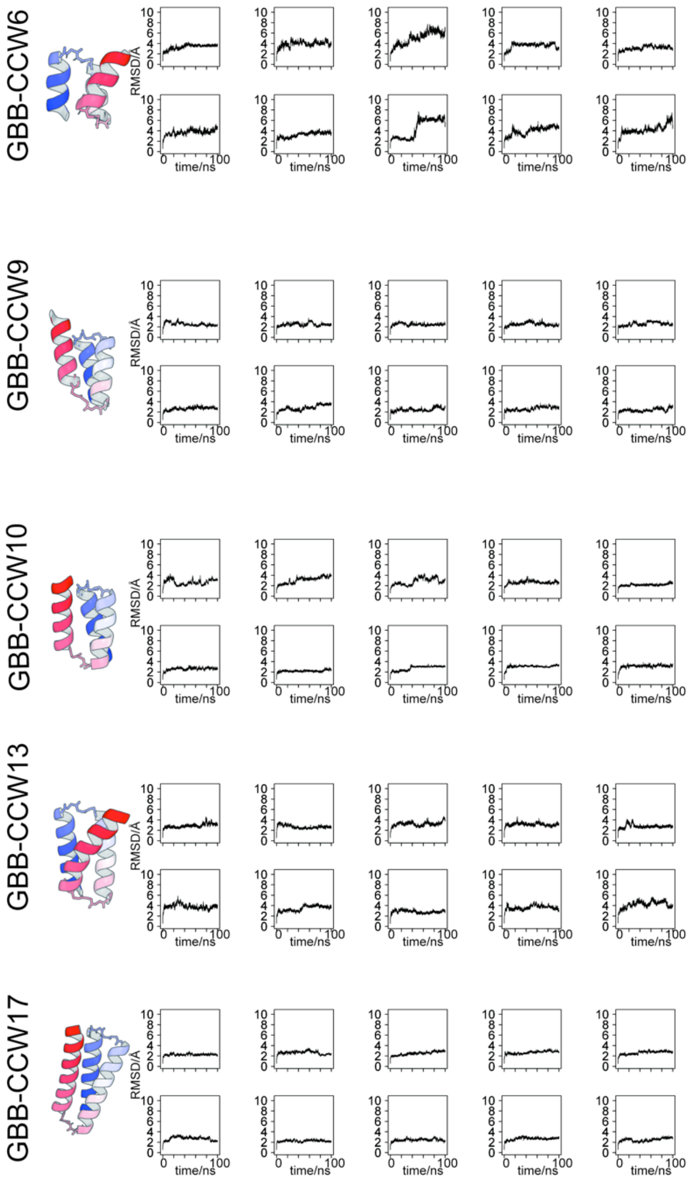

Figure S31 Time series of RMSD in 10 trajectories of 100 ns molecular dynamics simulations. (Left) Structure of designed proteins (Right) Time series of Cα RMSD referenced by the designed protein structure. The horizontal axis represents times in ns, and the veritcal axis represents RMSD in Å.

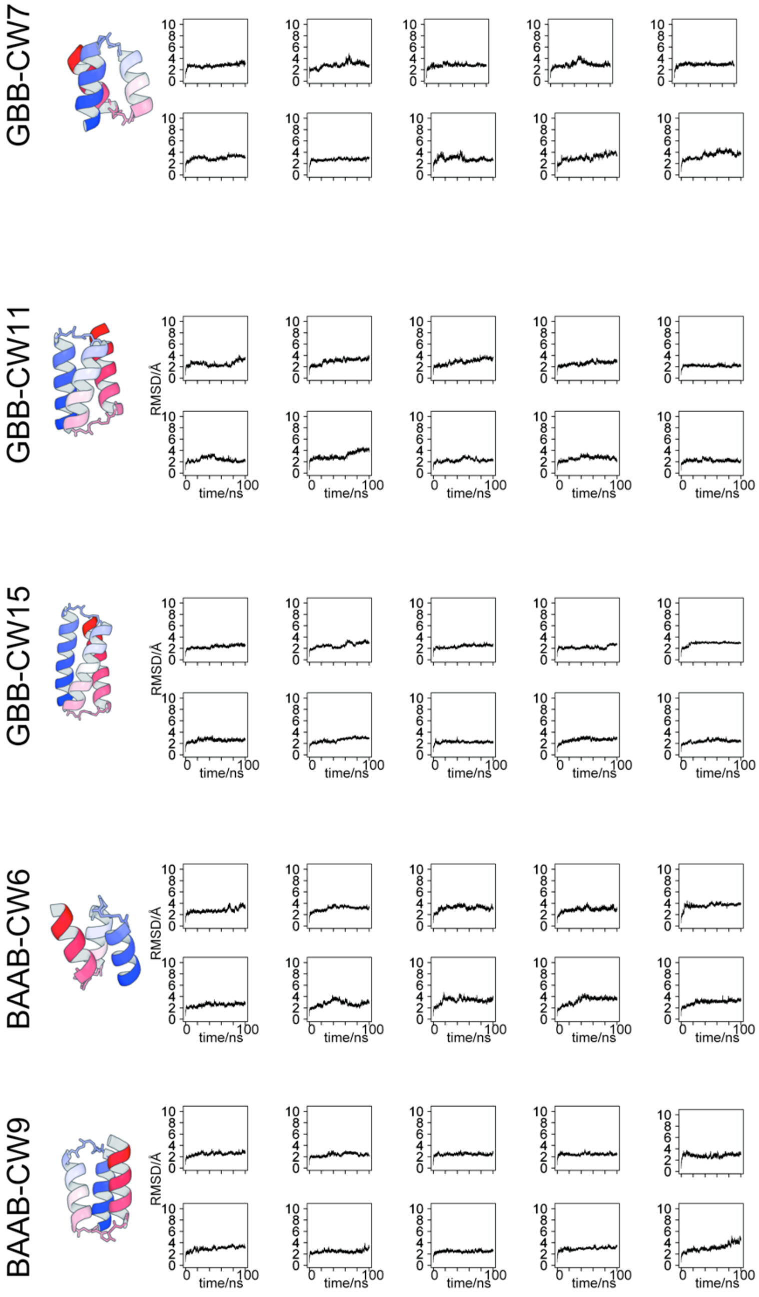

Figure S32: Time series of RMSD in 10 trajectories of 100 ns molecular dynamics simulations. (Left) Structure of designed proteins (Right) Time series of Cα RMSD referenced by the designed protein structure. The horizontal axis represents times in ns, and the veritcal axis represents RMSD in Å.

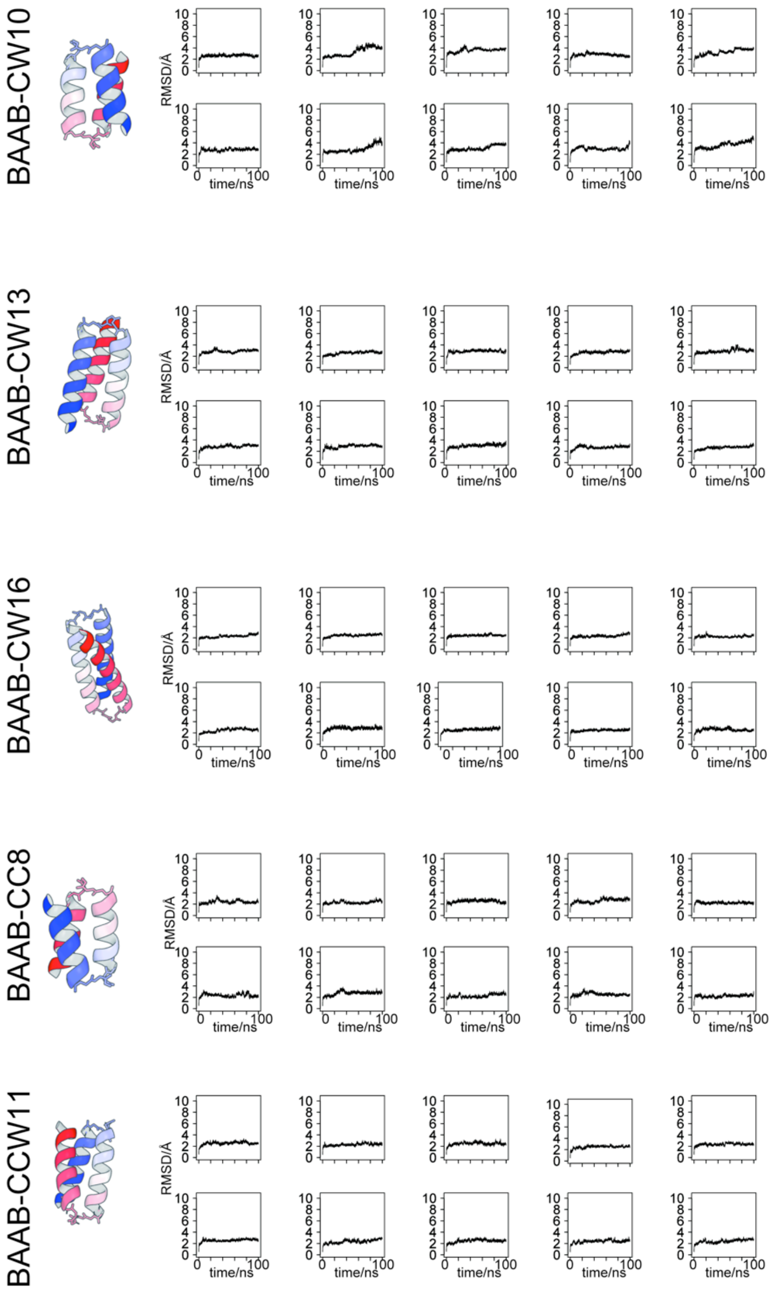

Figure S33: Time series of RMSD in 10 trajectories of 100 ns molecular dynamics simulations. (Left) Structure of designed proteins (Right) Time series of Cα RMSD referenced by the designed protein structure. The horizontal axis represents times in ns, and the veritcal axis represents RMSD in Å.

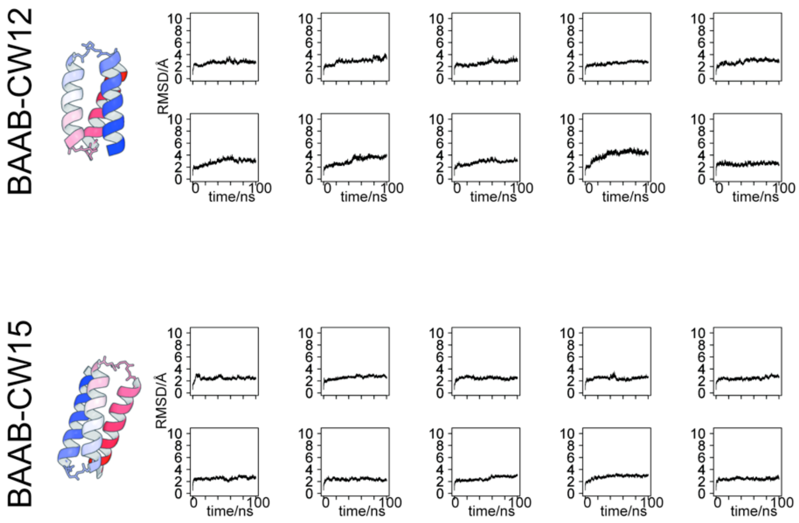

Figure S34: Time series of RMSD in 10 trajectories of 100 ns molecular dynamics simulations. (Left) Structure of designed proteins (Right) Time series of Cα RMSD referenced by the designed protein structure. The horizontal axis represents times in ns, and the veritcal axis represents RMSD in Å.

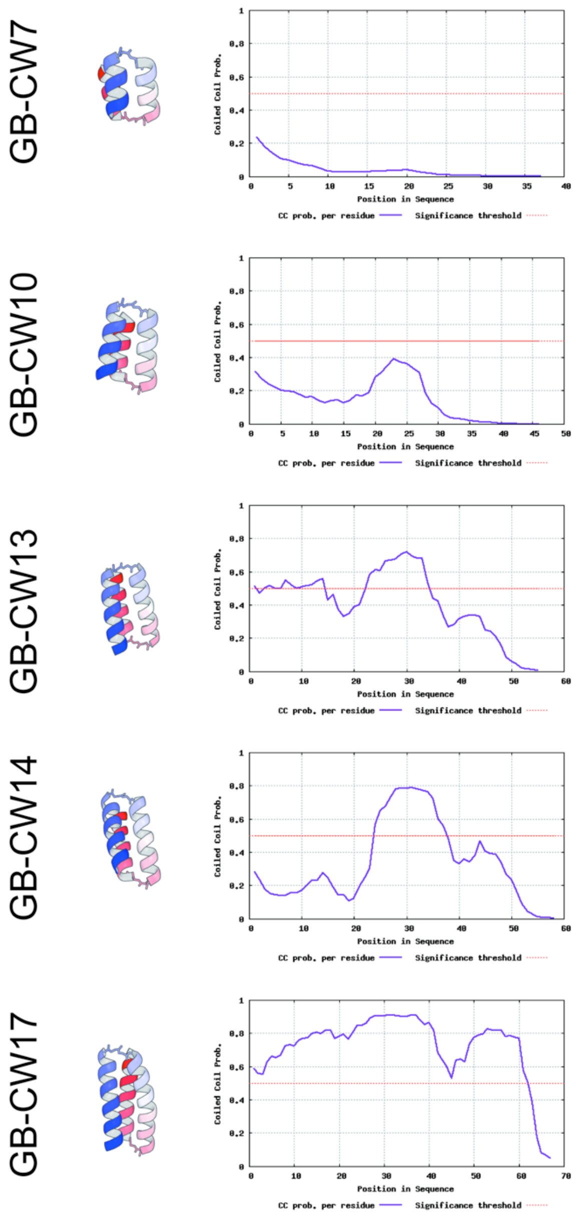

Figure S35: Probabilities that the designed sequence have coiled-coil arrangement of α-helix predicted by DeepCoil. (Left) Designed protein structures (Right) Predicted probability that the design sequences have coiled-coil arrangement of α-helix predicted by DeepCoil. The horizontal axis represents residue number, and the vertical axis represents the probability that the sequence is recognized as coiled-coil by DeepCoil.

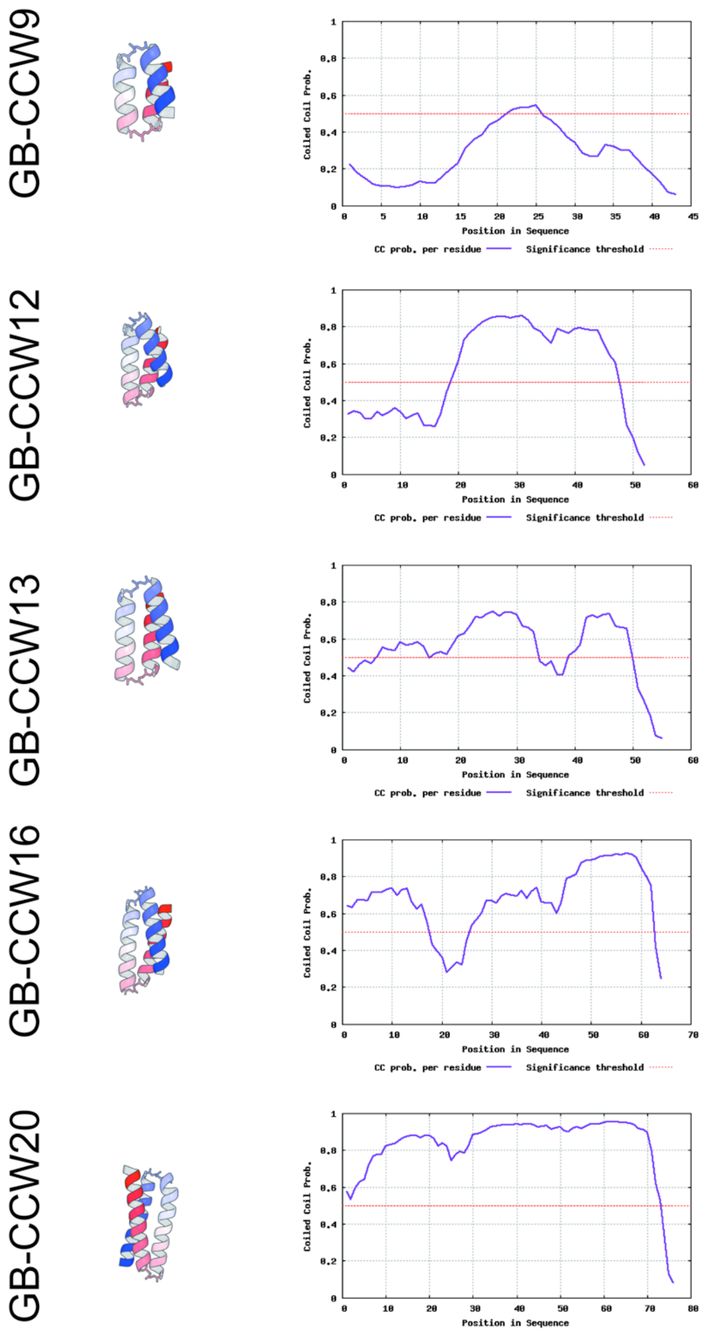

Figure S36: Probabilities that the designed sequence have coiled-coil arrangement of α-helix predicted by DeepCoil. (Left) Designed protein structures (Right) Predicted probability that the design sequences have coiled-coil arrangement of α-helix predicted by DeepCoil. The horizontal axis represents residue number, and the vertical axis represents the probability that the sequence is recognized as coiled-coil by DeepCoil.

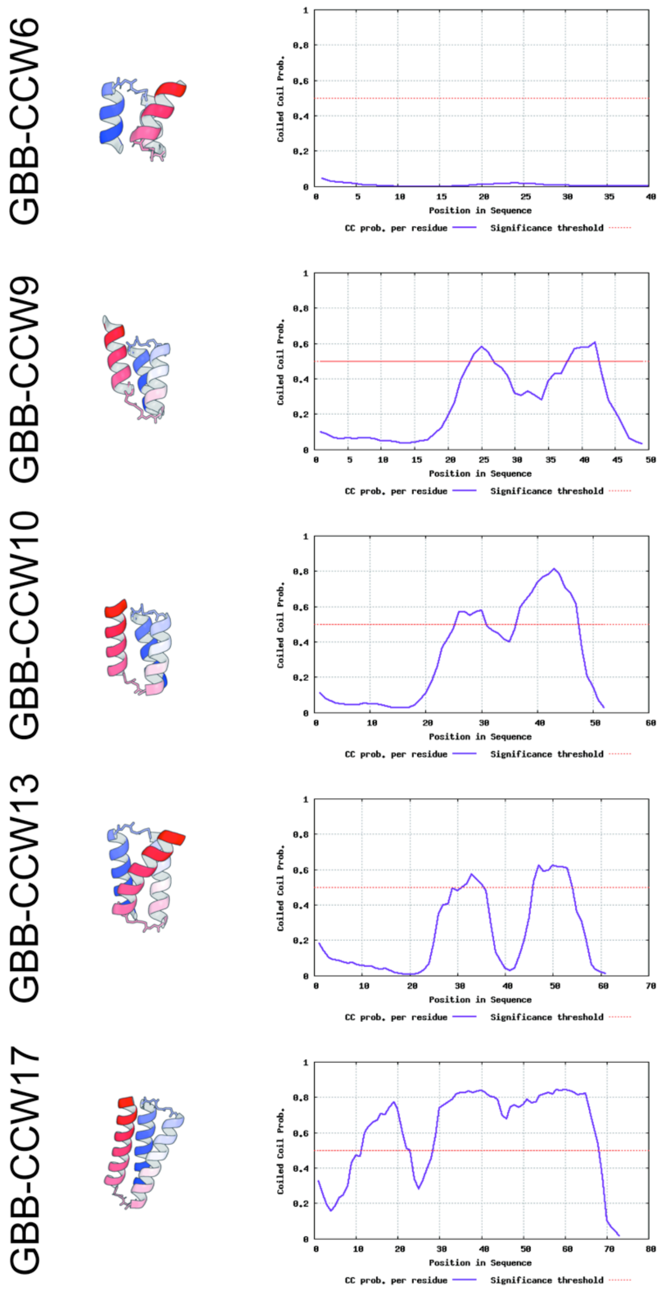

Figure S37: Probabilities that the designed sequence have coiled-coil arrangement of α-helix predicted by DeepCoil. (Left) Designed protein structures (Right) Predicted probability that the design sequences have coiled-coil arrangement of α-helix predicted by DeepCoil. The horizontal axis represents residue number, and the vertical axis represents the probability that the sequence is recognized as coiled-coil by DeepCoil.

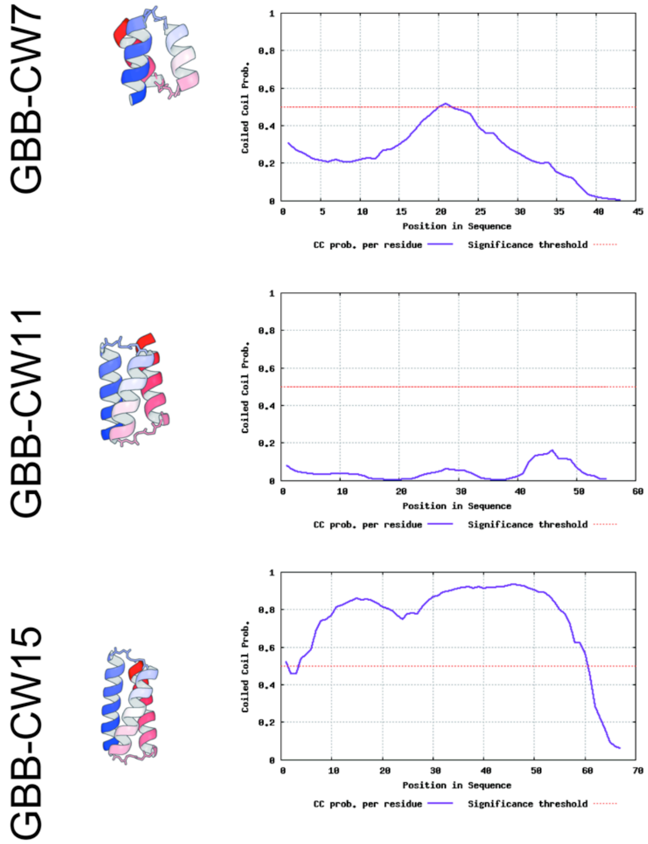

Figure S38: Probabilities that the designed sequence have coiled-coil arrangement of α-helix predicted by DeepCoil. (Left) Designed protein structures (Right) Predicted probability that the design sequences have coiled-coil arrangement of α-helix predicted by DeepCoil. The horizontal axis represents residue number, and the vertical axis represents the probability that the sequence is recognized as coiled-coil by DeepCoil.

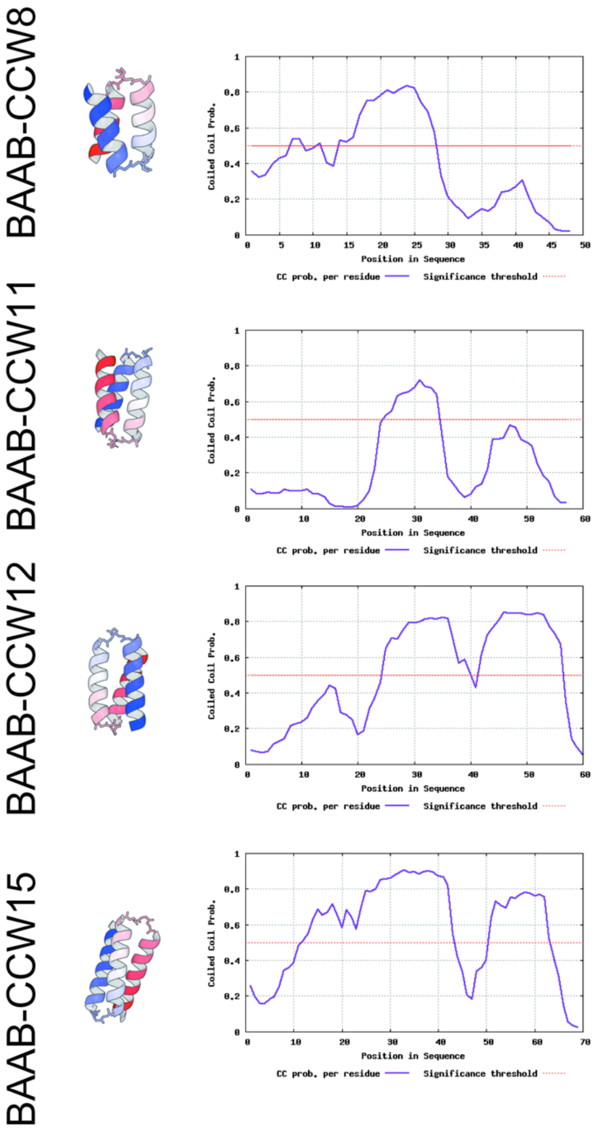

Figure S39: Probabilities that the designed sequence have coiled-coil arrangement of α-helix predicted by DeepCoil. (Left) Designed protein structures (Right) Predicted probability that the design sequences have coiled-coil arrangement of α-helix predicted by DeepCoil. The horizontal axis represents residue number, and the vertical axis represents the probability that the sequence is recognized as coiled-coil by DeepCoil.

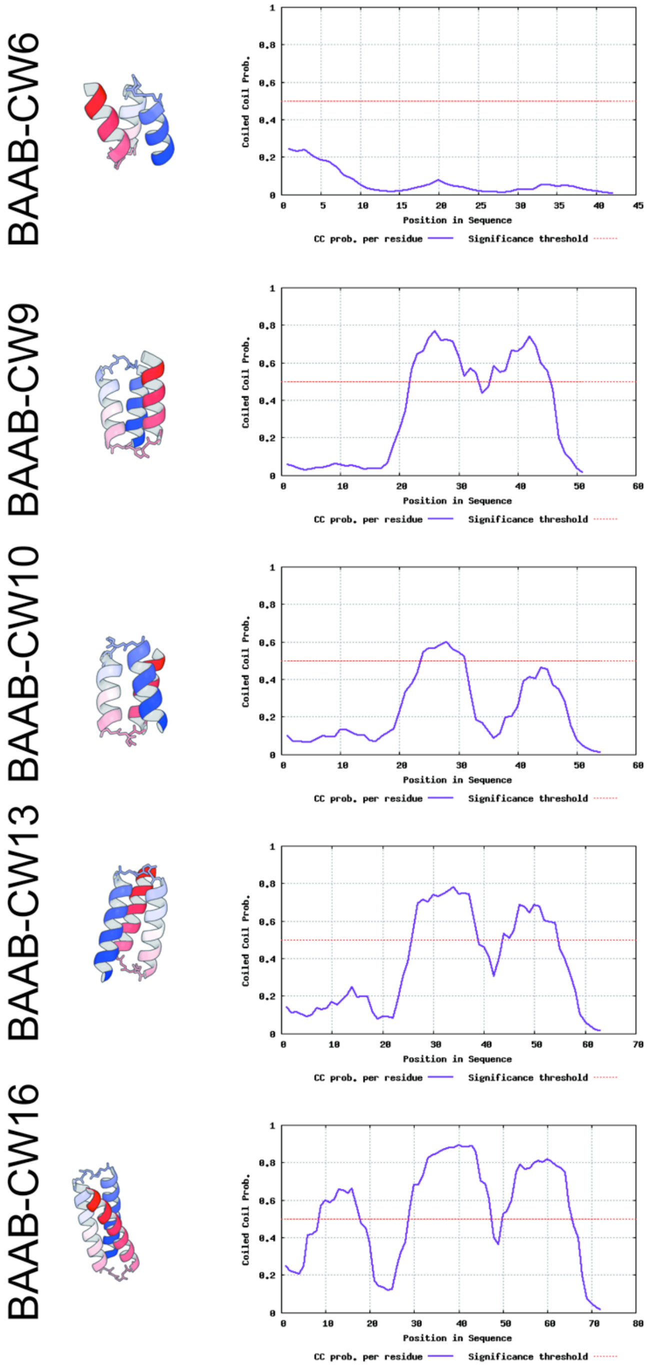

Figure S40: Probabilities that the designed sequence have coiled-coil arrangement of α-helix predicted by DeepCoil. (Left) Designed protein structures (Right) Predicted probability that the design sequences have coiled-coil arrangement of α-helix predicted by DeepCoil. The horizontal axis represents residue number, and the vertical axis represents the probability that the sequence is recognized as coiled-coil by DeepCoil.

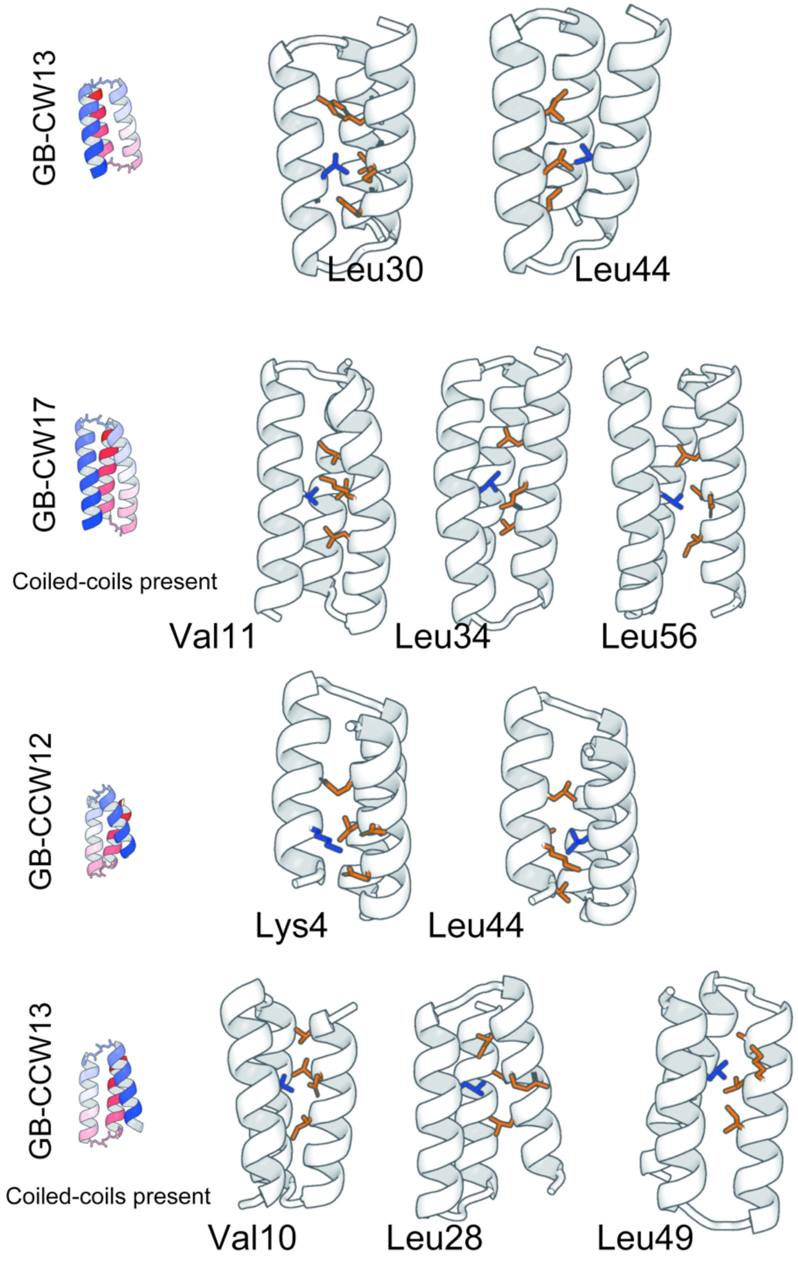

Figure S41: Knobs-into-holes in our design structures detected by SOCKET. (Left) Design structures (Right) Structure explaining knobs-into-holes sub-structures. The knob residues are colored in blue, and the hole residues are colored in orange. The residue name of knob residue is indicated below the structures.

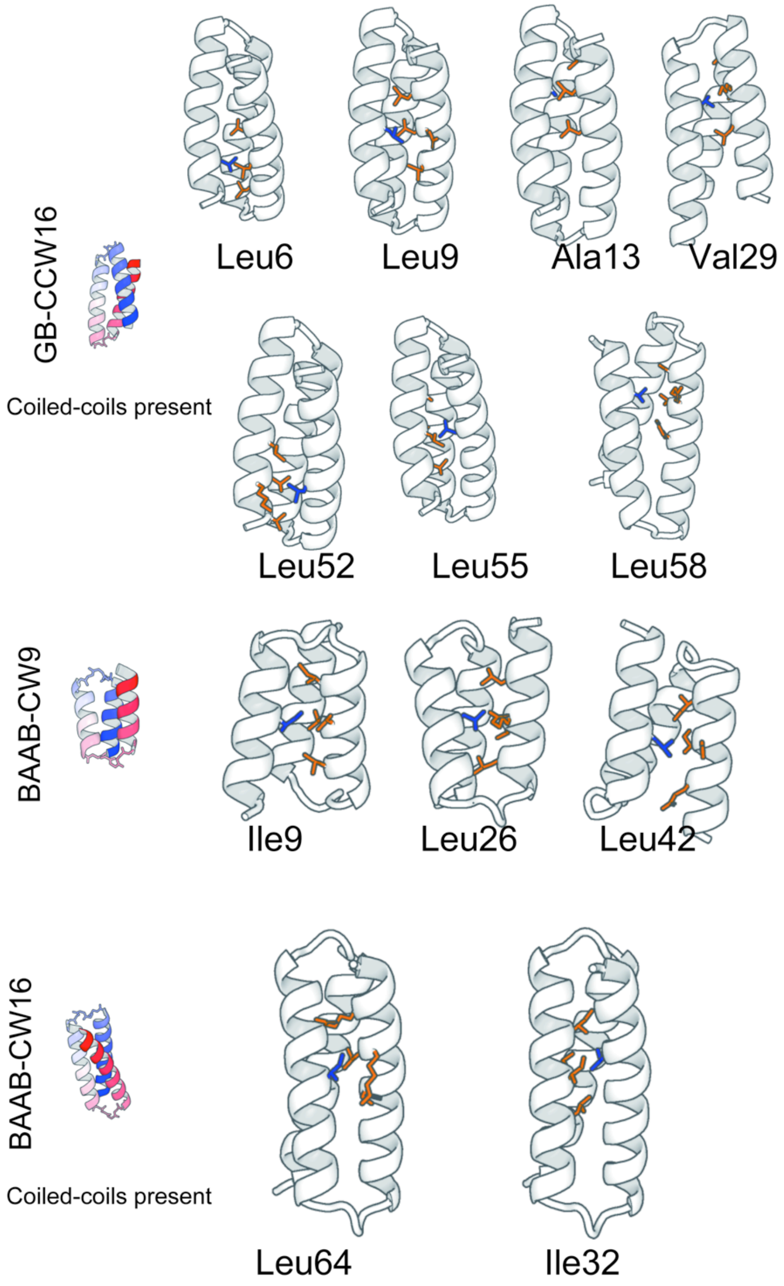

Figure S42: Knobs-into-holes in our design structures detected by SOCKET. (Left) Design structures (Right) Structure explaining knobs-into-holes sub-structures. The knob residues are colored in blue, and the hole residues are colored in orange. The residue name of knob residue is indicated below the structures.

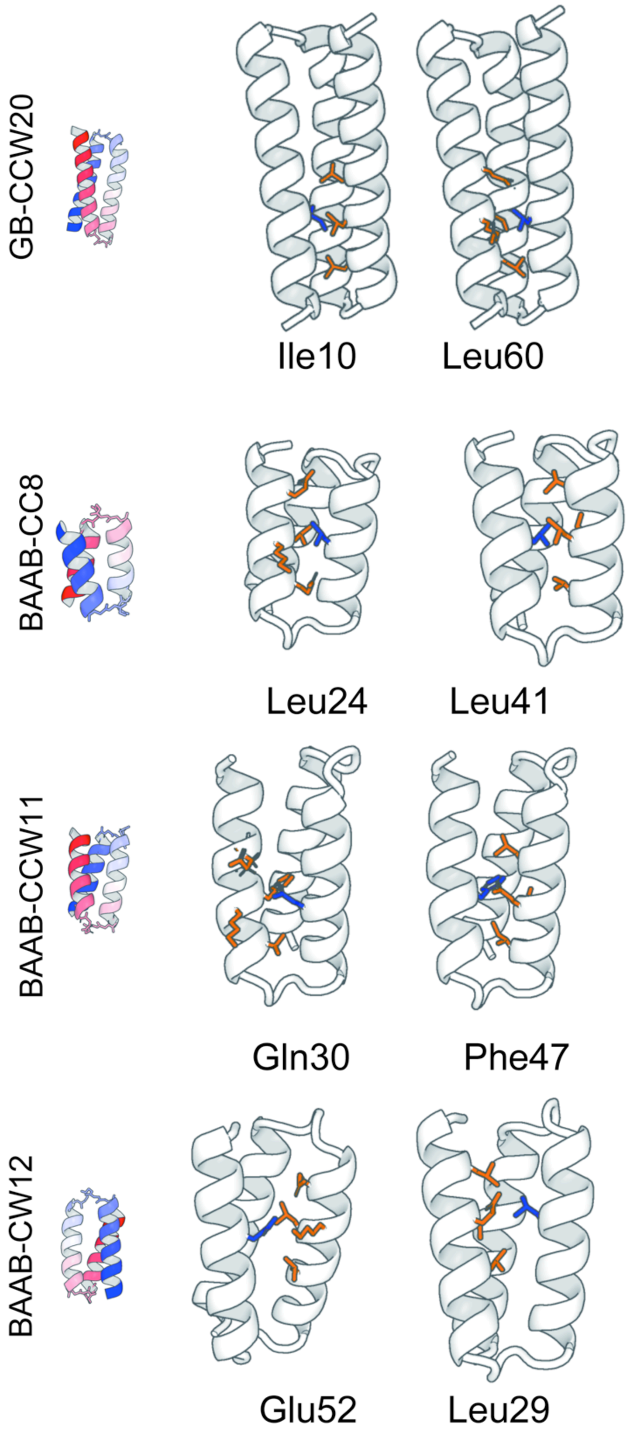

Figure S43: Knobs-into-holes in our design structures detected by SOCKET. (Left) Design structures (Right) Structure explaining knobs-into-holes sub-structures. The knob residues are colored in blue, and the hole residues are colored in orange. The residue name of knob residue is indicated below the structures.

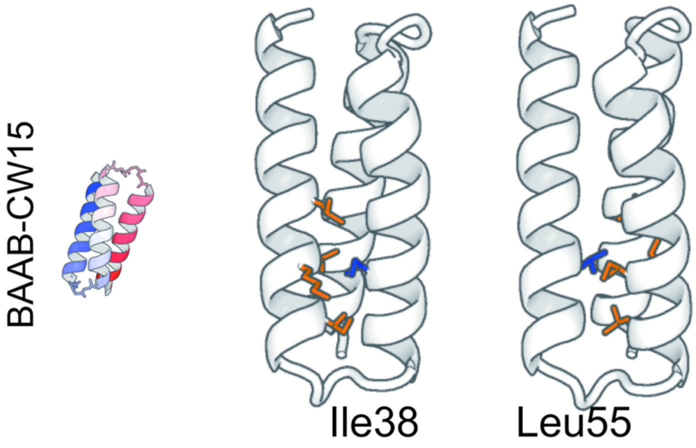

Figure S44: Knobs-into-holes in our design structures detected by SOCKET. (Left) Design structures (Right) Structure explaining knobs-into-holes sub-structures. The knob residues are colored in blue, and the hole residues are colored in orange. The residue name of knob residue is indicated below the structures.

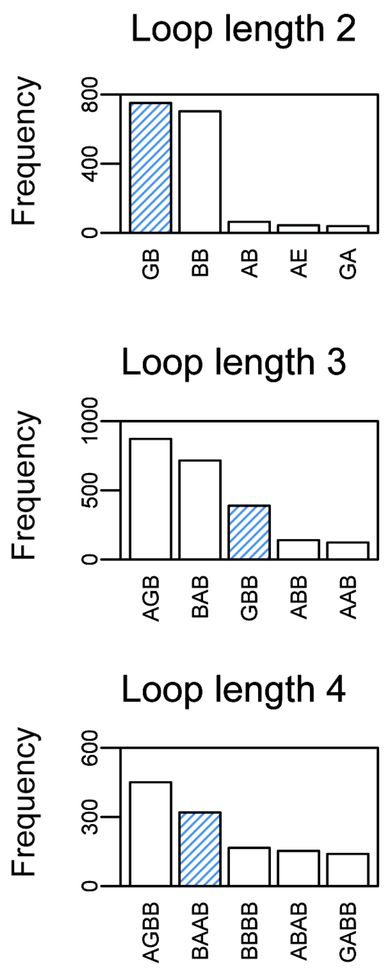

Figure S45. The population statistics of the hairpins in the ABEGO representation including loops starting/ending with A.

Structure	Loop 1	Loop 2
GB-CW7	GB (GB)	GB (GB)
GB-CW10	GB (GB)	GB (GB)
GB-CW13	GB (GB)	GBA (GB)
GB-CW14	GB (GB)	GB (GB)
GB-CW17	GB (GB)	GB (GB)
GB-CCW9	GB (GB)	GB (GB)
GB-CCW12	GB (GB)	GB (GB)
GB-CCW13	GB (GB)	AGB (GB)
GB-CCW16	GB (GB)	GB (GB)
GB-CCW20	GB (GB)	GB (GB)
GBB-CW7	GBB (GBB)	GBB (GBB)
GBB-CW11	GBB (GBB)	GBB (GBB)
GBB-CW15	GBB (GBB)	GBB (GBB)
GBB-CCW6	GBB (GBB)	GBB (GBB)
GBB-CCW9	GBB (GBB)	GBB (GBB)
GBB-CCW10	GBB (GBB)	GBB (GBB)
GBB-CCW13	GBB (GBB)	GBB (GBB)
GBB-CCW17	AGBB (GBB)	GBB (GBB)
BAAB-CW6	B (BAAB)	BAAB (BAAB)
BAAB-CW9	BAAB (BAAB)	BAAB (BAAB)
BAAB-CW10	BAAB (BAAB)	BAAB (BAAB)
BAAB-CW13	BAAB (BAAB)	BAAB (BAAB)
BAAB-CW16	BAAB (BAAB)	BAAB (BAAB)
BAAB-CCW8	BAAB (BAAB)	BAAB (BAAB)
BAAB-CCW11	BAAB (BAAB)	BAAB (BAAB)
BAAB-CCW12	BAAB (BAAB)	BAAB (BAAB)
BAAB-CCW15	BAAB (BAAB)	BAAB (BAAB)

Table S1: Comparison of backbone torsion angles between the lowest energy prediction structure from the sequence-dependent fragment assembly simulations for each design protein. The torsion angles observed in the lower energy structures are represented in the ABEGO representations, and their target torsion angles are represented in ABEGO in the parentheses. DSSP was used for assignment of secondary structure boundaries.

Most of the lowest energy structures recovered the same local conformations as design models. See also Figure S22—S26 for comparison of their overall topologies.

Structure	Mean score par residue (a.u.)
GB-CCW09	-2.38
GB-CCW12	-2.37
GB-CCW13	-2.41
GB-CCW16	-2.66
GB-CCW20	-2.61
GB-CW07	-2.28
GB-CW10	-2.04
GB-CW13	-2.36
GB-CW14	-2.66
GB-CW17	-2.65
GBB-CCW06	-2.25
GBB-CCW09	-2.27
GBB-CCW10	-2.33
GBB-CCW13	-2.49
GBB-CCW17	-2.57
GBB-CW07	-2.39
GBB-CW11	-2.42
GBB-CW15	-2.52
BAAB-CCW08	-2.38
BAAB-CCW11	-2.53
BAAB-CCW12	-2.38
BAAB-CCW15	-2.51
BAAB-CW06	-2.17
BAAB-CW09	-2.29
BAAB-CW10	-2.31
BAAB-CW13	-2.46
BAAB-CW16	-2.57
EE-CW	-2.00
EE-CCW	-2.11
BEB-CW	-2.08
BEB-CCW	-2.09
BEEE-CW	-2.15
BEEE-CCW	-2.02

Table S2: Mean Rosetta score par residue for best-effort design models. The structures were relaxed using Relax protocol of Rosetta and 1000 near-native structures were generated and their scores were calculated. Score Talaris2014 was used in the simulations and scoring. Scores were averaged over the 1000 structures and the number of residues of respective structures.
